# Study of the rare decays of $B^0_s$ and $B^0$ into muon pairs from data collected during the LHC Run 1 with the ATLAS detector

**DOI:** 10.1140/epjc/s10052-016-4338-8

**Published:** 2016-09-21

**Authors:** M. Aaboud, G. Aad, B. Abbott, J. Abdallah, O. Abdinov, B. Abeloos, R. Aben, O. S. AbouZeid, N. L. Abraham, H. Abramowicz, H. Abreu, R. Abreu, Y. Abulaiti, B. S. Acharya, L. Adamczyk, D. L. Adams, J. Adelman, S. Adomeit, T. Adye, A. A. Affolder, T. Agatonovic-Jovin, J. Agricola, J. A. Aguilar-Saavedra, S. P. Ahlen, F. Ahmadov, G. Aielli, H. Akerstedt, T. P. A. Åkesson, A. V. Akimov, G. L. Alberghi, J. Albert, S. Albrand, M. J. Alconada Verzini, M. Aleksa, I. N. Aleksandrov, C. Alexa, G. Alexander, T. Alexopoulos, M. Alhroob, M. Aliev, G. Alimonti, J. Alison, S. P. Alkire, B. M. M. Allbrooke, B. W. Allen, P. P. Allport, A. Aloisio, A. Alonso, F. Alonso, C. Alpigiani, M. Alstaty, B. Alvarez Gonzalez, D. Álvarez Piqueras, M. G. Alviggi, B. T. Amadio, K. Amako, Y. Amaral Coutinho, C. Amelung, D. Amidei, S. P. Amor Dos Santos, A. Amorim, S. Amoroso, G. Amundsen, C. Anastopoulos, L. S. Ancu, N. Andari, T. Andeen, C. F. Anders, G. Anders, J. K. Anders, K. J. Anderson, A. Andreazza, V. Andrei, S. Angelidakis, I. Angelozzi, P. Anger, A. Angerami, F. Anghinolfi, A. V. Anisenkov, N. Anjos, A. Annovi, M. Antonelli, A. Antonov, F. Anulli, M. Aoki, L. Aperio Bella, G. Arabidze, Y. Arai, J. P. Araque, A. T. H. Arce, F. A. Arduh, J-F. Arguin, S. Argyropoulos, M. Arik, A. J. Armbruster, L. J. Armitage, O. Arnaez, H. Arnold, M. Arratia, O. Arslan, A. Artamonov, G. Artoni, S. Artz, S. Asai, N. Asbah, A. Ashkenazi, B. Åsman, L. Asquith, K. Assamagan, R. Astalos, M. Atkinson, N. B. Atlay, K. Augsten, G. Avolio, B. Axen, M. K. Ayoub, G. Azuelos, M. A. Baak, A. E. Baas, M. J. Baca, H. Bachacou, K. Bachas, M. Backes, M. Backhaus, P. Bagiacchi, P. Bagnaia, Y. Bai, J. T. Baines, O. K. Baker, E. M. Baldin, P. Balek, T. Balestri, F. Balli, W. K. Balunas, E. Banas, Sw. Banerjee, A. A. E. Bannoura, L. Barak, E. L. Barberio, D. Barberis, M. Barbero, T. Barillari, T. Barklow, N. Barlow, S. L. Barnes, B. M. Barnett, R. M. Barnett, Z. Barnovska, A. Baroncelli, G. Barone, A. J. Barr, L. Barranco Navarro, F. Barreiro, J. Barreiro Guimarães da Costa, R. Bartoldus, A. E. Barton, P. Bartos, A. Basalaev, A. Bassalat, R. L. Bates, S. J. Batista, J. R. Batley, M. Battaglia, M. Bauce, F. Bauer, H. S. Bawa, J. B. Beacham, M. D. Beattie, T. Beau, P. H. Beauchemin, P. Bechtle, H. P. Beck, K. Becker, M. Becker, M. Beckingham, C. Becot, A. J. Beddall, A. Beddall, V. A. Bednyakov, M. Bedognetti, C. P. Bee, L. J. Beemster, T. A. Beermann, M. Begel, J. K. Behr, C. Belanger-Champagne, A. S. Bell, G. Bella, L. Bellagamba, A. Bellerive, M. Bellomo, K. Belotskiy, O. Beltramello, N. L. Belyaev, O. Benary, D. Benchekroun, M. Bender, K. Bendtz, N. Benekos, Y. Benhammou, E. Benhar Noccioli, J. Benitez, D. P. Benjamin, J. R. Bensinger, S. Bentvelsen, L. Beresford, M. Beretta, D. Berge, E. Bergeaas Kuutmann, N. Berger, J. Beringer, S. Berlendis, N. R. Bernard, C. Bernius, F. U. Bernlochner, T. Berry, P. Berta, C. Bertella, G. Bertoli, F. Bertolucci, I. A. Bertram, C. Bertsche, D. Bertsche, G. J. Besjes, O. Bessidskaia Bylund, M. Bessner, N. Besson, C. Betancourt, S. Bethke, A. J. Bevan, W. Bhimji, R. M. Bianchi, L. Bianchini, M. Bianco, O. Biebel, D. Biedermann, R. Bielski, N. V. Biesuz, M. Biglietti, J. Bilbao De Mendizabal, H. Bilokon, M. Bindi, S. Binet, A. Bingul, C. Bini, S. Biondi, D. M. Bjergaard, C. W. Black, J. E. Black, K. M. Black, D. Blackburn, R. E. Blair, J. -B. Blanchard, J. E. Blanco, T. Blazek, I. Bloch, C. Blocker, W. Blum, U. Blumenschein, S. Blunier, G. J. Bobbink, V. S. Bobrovnikov, S. S. Bocchetta, A. Bocci, C. Bock, M. Boehler, D. Boerner, J. A. Bogaerts, D. Bogavac, A. G. Bogdanchikov, C. Bohm, V. Boisvert, P. Bokan, T. Bold, A. S. Boldyrev, M. Bomben, M. Bona, M. Boonekamp, A. Borisov, G. Borissov, J. Bortfeldt, D. Bortoletto, V. Bortolotto, K. Bos, D. Boscherini, M. Bosman, J. D. Bossio Sola, J. Boudreau, J. Bouffard, E. V. Bouhova-Thacker, D. Boumediene, C. Bourdarios, S. K. Boutle, A. Boveia, J. Boyd, I. R. Boyko, J. Bracinik, A. Brandt, G. Brandt, O. Brandt, U. Bratzler, B. Brau, J. E. Brau, H. M. Braun, W. D. Breaden Madden, K. Brendlinger, A. J. Brennan, L. Brenner, R. Brenner, S. Bressler, T. M. Bristow, D. Britton, D. Britzger, F. M. Brochu, I. Brock, R. Brock, G. Brooijmans, T. Brooks, W. K. Brooks, J. Brosamer, E. Brost, J. H Broughton, P. A. Bruckman de Renstrom, D. Bruncko, R. Bruneliere, A. Bruni, G. Bruni, BH Brunt, M. Bruschi, N. Bruscino, P. Bryant, L. Bryngemark, T. Buanes, Q. Buat, P. Buchholz, A. G. Buckley, I. A. Budagov, F. Buehrer, M. K. Bugge, O. Bulekov, D. Bullock, H. Burckhart, S. Burdin, C. D. Burgard, B. Burghgrave, K. Burka, S. Burke, I. Burmeister, E. Busato, D. Büscher, V. Büscher, P. Bussey, J. M. Butler, C. M. Buttar, J. M. Butterworth, P. Butti, W. Buttinger, A. Buzatu, A. R. Buzykaev, S. Cabrera Urbán, D. Caforio, V. M. Cairo, O. Cakir, N. Calace, P. Calafiura, A. Calandri, G. Calderini, P. Calfayan, L. P. Caloba, D. Calvet, S. Calvet, T. P. Calvet, R. Camacho Toro, S. Camarda, P. Camarri, D. Cameron, R. Caminal Armadans, C. Camincher, S. Campana, M. Campanelli, A. Camplani, A. Campoverde, V. Canale, A. Canepa, M. Cano Bret, J. Cantero, R. Cantrill, T. Cao, M. D. M. Capeans Garrido, I. Caprini, M. Caprini, M. Capua, R. Caputo, R. M. Carbone, R. Cardarelli, F. Cardillo, I. Carli, T. Carli, G. Carlino, L. Carminati, S. Caron, E. Carquin, G. D. Carrillo-Montoya, J. R. Carter, J. Carvalho, D. Casadei, M. P. Casado, M. Casolino, D. W. Casper, E. Castaneda-Miranda, R. Castelijn, A. Castelli, V. Castillo Gimenez, N. F. Castro, A. Catinaccio, J. R. Catmore, A. Cattai, J. Caudron, V. Cavaliere, E. Cavallaro, D. Cavalli, M. Cavalli-Sforza, V. Cavasinni, F. Ceradini, L. Cerda Alberich, B. C. Cerio, A. S. Cerqueira, A. Cerri, L. Cerrito, F. Cerutti, M. Cerv, A. Cervelli, S. A. Cetin, A. Chafaq, D. Chakraborty, S. K. Chan, Y. L. Chan, P. Chang, J. D. Chapman, D. G. Charlton, A. Chatterjee, C. C. Chau, C. A. Chavez Barajas, S. Che, S. Cheatham, A. Chegwidden, S. Chekanov, S. V. Chekulaev, G. A. Chelkov, M. A. Chelstowska, C. Chen, H. Chen, K. Chen, S. Chen, S. Chen, X. Chen, Y. Chen, H. C. Cheng, H. J Cheng, Y. Cheng, A. Cheplakov, E. Cheremushkina, R. Cherkaoui El Moursli, V. Chernyatin, E. Cheu, L. Chevalier, V. Chiarella, G. Chiarelli, G. Chiodini, A. S. Chisholm, A. Chitan, M. V. Chizhov, K. Choi, A. R. Chomont, S. Chouridou, B. K. B. Chow, V. Christodoulou, D. Chromek-Burckhart, J. Chudoba, A. J. Chuinard, J. J. Chwastowski, L. Chytka, G. Ciapetti, A. K. Ciftci, D. Cinca, V. Cindro, I. A. Cioara, A. Ciocio, F. Cirotto, Z. H. Citron, M. Citterio, M. Ciubancan, A. Clark, B. L. Clark, M. R. Clark, P. J. Clark, R. N. Clarke, C. Clement, Y. Coadou, M. Cobal, A. Coccaro, J. Cochran, L. Coffey, L. Colasurdo, B. Cole, A. P. Colijn, J. Collot, T. Colombo, G. Compostella, P. Conde Muiño, E. Coniavitis, S. H. Connell, I. A. Connelly, V. Consorti, S. Constantinescu, G. Conti, F. Conventi, M. Cooke, B. D. Cooper, A. M. Cooper-Sarkar, K. J. R. Cormier, T. Cornelissen, M. Corradi, F. Corriveau, A. Corso-Radu, A. Cortes-Gonzalez, G. Cortiana, G. Costa, M. J. Costa, D. Costanzo, G. Cottin, G. Cowan, B. E. Cox, K. Cranmer, S. J. Crawley, G. Cree, S. Crépé-Renaudin, F. Crescioli, W. A. Cribbs, M. Crispin Ortuzar, M. Cristinziani, V. Croft, G. Crosetti, T. Cuhadar Donszelmann, J. Cummings, M. Curatolo, J. Cúth, C. Cuthbert, H. Czirr, P. Czodrowski, G. D’amen, S. D’Auria, M. D’Onofrio, M. J. Da Cunha Sargedas De Sousa, C. Da Via, W. Dabrowski, T. Dado, T. Dai, O. Dale, F. Dallaire, C. Dallapiccola, M. Dam, J. R. Dandoy, N. P. Dang, A. C. Daniells, N. S. Dann, M. Danninger, M. Dano Hoffmann, V. Dao, G. Darbo, S. Darmora, J. Dassoulas, A. Dattagupta, W. Davey, C. David, T. Davidek, M. Davies, P. Davison, E. Dawe, I. Dawson, R. K. Daya-Ishmukhametova, K. De, R. de Asmundis, A. De Benedetti, S. De Castro, S. De Cecco, N. De Groot, P. de Jong, H. De la Torre, F. De Lorenzi, A. De Maria, D. De Pedis, A. De Salvo, U. De Sanctis, A. De Santo, J. B. De Vivie De Regie, W. J. Dearnaley, R. Debbe, C. Debenedetti, D. V. Dedovich, N. Dehghanian, I. Deigaard, M. Del Gaudio, J. Del Peso, T. Del Prete, D. Delgove, F. Deliot, C. M. Delitzsch, M. Deliyergiyev, A. Dell’Acqua, L. Dell’Asta, M. Dell’Orso, M. Della Pietra, D. della Volpe, M. Delmastro, P. A. Delsart, C. Deluca, D. A. DeMarco, S. Demers, M. Demichev, A. Demilly, S. P. Denisov, D. Denysiuk, D. Derendarz, J. E. Derkaoui, F. Derue, P. Dervan, K. Desch, C. Deterre, K. Dette, P. O. Deviveiros, A. Dewhurst, S. Dhaliwal, A. Di Ciaccio, L. Di Ciaccio, W. K. Di Clemente, C. Di Donato, A. Di Girolamo, B. Di Girolamo, B. Di Micco, R. Di Nardo, A. Di Simone, R. Di Sipio, D. Di Valentino, C. Diaconu, M. Diamond, F. A. Dias, M. A. Diaz, E. B. Diehl, J. Dietrich, S. Diglio, A. Dimitrievska, J. Dingfelder, P. Dita, S. Dita, F. Dittus, F. Djama, T. Djobava, J. I. Djuvsland, M. A. B. do Vale, D. Dobos, M. Dobre, C. Doglioni, T. Dohmae, J. Dolejsi, Z. Dolezal, B. A. Dolgoshein, M. Donadelli, S. Donati, P. Dondero, J. Donini, J. Dopke, A. Doria, M. T. Dova, A. T. Doyle, E. Drechsler, M. Dris, Y. Du, J. Duarte-Campderros, E. Duchovni, G. Duckeck, O. A. Ducu, D. Duda, A. Dudarev, E. M. Duffield, L. Duflot, L. Duguid, M. Dührssen, M. Dumancic, M. Dunford, H. Duran Yildiz, M. Düren, A. Durglishvili, D. Duschinger, B. Dutta, M. Dyndal, C. Eckardt, K. M. Ecker, R. C. Edgar, N. C. Edwards, T. Eifert, G. Eigen, K. Einsweiler, T. Ekelof, M. El Kacimi, V. Ellajosyula, M. Ellert, S. Elles, F. Ellinghaus, A. A. Elliot, N. Ellis, J. Elmsheuser, M. Elsing, D. Emeliyanov, Y. Enari, O. C. Endner, M. Endo, J. S. Ennis, J. Erdmann, A. Ereditato, G. Ernis, J. Ernst, M. Ernst, S. Errede, E. Ertel, M. Escalier, H. Esch, C. Escobar, B. Esposito, A. I. Etienvre, E. Etzion, H. Evans, A. Ezhilov, F. Fabbri, L. Fabbri, G. Facini, R. M. Fakhrutdinov, S. Falciano, R. J. Falla, J. Faltova, Y. Fang, M. Fanti, A. Farbin, A. Farilla, C. Farina, T. Farooque, S. Farrell, S. M. Farrington, P. Farthouat, F. Fassi, P. Fassnacht, D. Fassouliotis, M. Faucci Giannelli, A. Favareto, W. J. Fawcett, L. Fayard, O. L. Fedin, W. Fedorko, S. Feigl, L. Feligioni, C. Feng, E. J. Feng, H. Feng, A. B. Fenyuk, L. Feremenga, P. Fernandez Martinez, S. Fernandez Perez, J. Ferrando, A. Ferrari, P. Ferrari, R. Ferrari, D. E. Ferreira de Lima, A. Ferrer, D. Ferrere, C. Ferretti, A. Ferretto Parodi, F. Fiedler, A. Filipčič, M. Filipuzzi, F. Filthaut, M. Fincke-Keeler, K. D. Finelli, M. C. N. Fiolhais, L. Fiorini, A. Firan, A. Fischer, C. Fischer, J. Fischer, W. C. Fisher, N. Flaschel, I. Fleck, P. Fleischmann, G. T. Fletcher, R. R. M. Fletcher, T. Flick, A. Floderus, L. R. Flores Castillo, M. J. Flowerdew, G. T. Forcolin, A. Formica, A. Forti, A. G. Foster, D. Fournier, H. Fox, S. Fracchia, P. Francavilla, M. Franchini, D. Francis, L. Franconi, M. Franklin, M. Frate, M. Fraternali, D. Freeborn, S. M. Fressard-Batraneanu, F. Friedrich, D. Froidevaux, J. A. Frost, C. Fukunaga, E. Fullana Torregrosa, T. Fusayasu, J. Fuster, C. Gabaldon, O. Gabizon, A. Gabrielli, A. Gabrielli, G. P. Gach, S. Gadatsch, S. Gadomski, G. Gagliardi, L. G. Gagnon, P. Gagnon, C. Galea, B. Galhardo, E. J. Gallas, B. J. Gallop, P. Gallus, G. Galster, K. K. Gan, J. Gao, Y. Gao, Y. S. Gao, F. M. Garay Walls, C. García, J. E. García Navarro, M. Garcia-Sciveres, R. W. Gardner, N. Garelli, V. Garonne, A. Gascon Bravo, C. Gatti, A. Gaudiello, G. Gaudio, B. Gaur, L. Gauthier, I. L. Gavrilenko, C. Gay, G. Gaycken, E. N. Gazis, Z. Gecse, C. N. P. Gee, Ch. Geich-Gimbel, M. Geisen, M. P. Geisler, C. Gemme, M. H. Genest, C. Geng, S. Gentile, S. George, D. Gerbaudo, A. Gershon, S. Ghasemi, H. Ghazlane, M. Ghneimat, B. Giacobbe, S. Giagu, P. Giannetti, B. Gibbard, S. M. Gibson, M. Gignac, M. Gilchriese, T. P. S. Gillam, D. Gillberg, G. Gilles, D. M. Gingrich, N. Giokaris, M. P. Giordani, F. M. Giorgi, F. M. Giorgi, P. F. Giraud, P. Giromini, D. Giugni, F. Giuli, C. Giuliani, M. Giulini, B. K. Gjelsten, S. Gkaitatzis, I. Gkialas, E. L. Gkougkousis, L. K. Gladilin, C. Glasman, J. Glatzer, P. C. F. Glaysher, A. Glazov, M. Goblirsch-Kolb, J. Godlewski, S. Goldfarb, T. Golling, D. Golubkov, A. Gomes, R. Gonçalo, J. Goncalves Pinto Firmino Da Costa, L. Gonella, A. Gongadze, S. González de la Hoz, G. Gonzalez Parra, S. Gonzalez-Sevilla, L. Goossens, P. A. Gorbounov, H. A. Gordon, I. Gorelov, B. Gorini, E. Gorini, A. Gorišek, E. Gornicki, A. T. Goshaw, C. Gössling, M. I. Gostkin, C. R. Goudet, D. Goujdami, A. G. Goussiou, N. Govender, E. Gozani, L. Graber, I. Grabowska-Bold, P. O. J. Gradin, P. Grafström, J. Gramling, E. Gramstad, S. Grancagnolo, V. Gratchev, P. M. Gravila, H. M. Gray, E. Graziani, Z. D. Greenwood, C. Grefe, K. Gregersen, I. M. Gregor, P. Grenier, K. Grevtsov, J. Griffiths, A. A. Grillo, K. Grimm, S. Grinstein, Ph. Gris, J. -F. Grivaz, S. Groh, J. P. Grohs, E. Gross, J. Grosse-Knetter, G. C. Grossi, Z. J. Grout, L. Guan, W. Guan, J. Guenther, F. Guescini, D. Guest, O. Gueta, E. Guido, T. Guillemin, S. Guindon, U. Gul, C. Gumpert, J. Guo, Y. Guo, S. Gupta, G. Gustavino, P. Gutierrez, N. G. Gutierrez Ortiz, C. Gutschow, C. Guyot, C. Gwenlan, C. B. Gwilliam, A. Haas, C. Haber, H. K. Hadavand, N. Haddad, A. Hadef, P. Haefner, S. Hageböck, Z. Hajduk, H. Hakobyan, M. Haleem, J. Haley, G. Halladjian, G. D. Hallewell, K. Hamacher, P. Hamal, K. Hamano, A. Hamilton, G. N. Hamity, P. G. Hamnett, L. Han, K. Hanagaki, K. Hanawa, M. Hance, B. Haney, P. Hanke, R. Hanna, J. B. Hansen, J. D. Hansen, M. C. Hansen, P. H. Hansen, K. Hara, A. S. Hard, T. Harenberg, F. Hariri, S. Harkusha, R. D. Harrington, P. F. Harrison, F. Hartjes, N. M. Hartmann, M. Hasegawa, Y. Hasegawa, A. Hasib, S. Hassani, S. Haug, R. Hauser, L. Hauswald, M. Havranek, C. M. Hawkes, R. J. Hawkings, D. Hayden, C. P. Hays, J. M. Hays, H. S. Hayward, S. J. Haywood, S. J. Head, T. Heck, V. Hedberg, L. Heelan, S. Heim, T. Heim, B. Heinemann, J. J. Heinrich, L. Heinrich, C. Heinz, J. Hejbal, L. Helary, S. Hellman, C. Helsens, J. Henderson, R. C. W. Henderson, Y. Heng, S. Henkelmann, A. M. Henriques Correia, S. Henrot-Versille, G. H. Herbert, Y. Hernández Jiménez, G. Herten, R. Hertenberger, L. Hervas, G. G. Hesketh, N. P. Hessey, J. W. Hetherly, R. Hickling, E. Higón-Rodriguez, E. Hill, J. C. Hill, K. H. Hiller, S. J. Hillier, I. Hinchliffe, E. Hines, R. R. Hinman, M. Hirose, D. Hirschbuehl, J. Hobbs, N. Hod, M. C. Hodgkinson, P. Hodgson, A. Hoecker, M. R. Hoeferkamp, F. Hoenig, D. Hohn, T. R. Holmes, M. Homann, T. M. Hong, B. H. Hooberman, W. H. Hopkins, Y. Horii, A. J. Horton, J-Y. Hostachy, S. Hou, A. Hoummada, J. Howarth, M. Hrabovsky, I. Hristova, J. Hrivnac, T. Hryn’ova, A. Hrynevich, C. Hsu, P. J. Hsu, S. -C. Hsu, D. Hu, Q. Hu, Y. Huang, Z. Hubacek, F. Hubaut, F. Huegging, T. B. Huffman, E. W. Hughes, G. Hughes, M. Huhtinen, T. A. Hülsing, P. Huo, N. Huseynov, J. Huston, J. Huth, G. Iacobucci, G. Iakovidis, I. Ibragimov, L. Iconomidou-Fayard, E. Ideal, Z. Idrissi, P. Iengo, O. Igonkina, T. Iizawa, Y. Ikegami, M. Ikeno, Y. Ilchenko, D. Iliadis, N. Ilic, T. Ince, G. Introzzi, P. Ioannou, M. Iodice, K. Iordanidou, V. Ippolito, M. Ishino, M. Ishitsuka, R. Ishmukhametov, C. Issever, S. Istin, F. Ito, J. M. Iturbe Ponce, R. Iuppa, W. Iwanski, H. Iwasaki, J. M. Izen, V. Izzo, S. Jabbar, B. Jackson, M. Jackson, P. Jackson, V. Jain, K. B. Jakobi, K. Jakobs, S. Jakobsen, T. Jakoubek, D. O. Jamin, D. K. Jana, E. Jansen, R. Jansky, J. Janssen, M. Janus, G. Jarlskog, N. Javadov, T. Javůrek, F. Jeanneau, L. Jeanty, J. Jejelava, G. -Y. Jeng, D. Jennens, P. Jenni, J. Jentzsch, C. Jeske, S. Jézéquel, H. Ji, J. Jia, H. Jiang, Y. Jiang, S. Jiggins, J. Jimenez Pena, S. Jin, A. Jinaru, O. Jinnouchi, P. Johansson, K. A. Johns, W. J. Johnson, K. Jon-And, G. Jones, R. W. L. Jones, S. Jones, T. J. Jones, J. Jongmanns, P. M. Jorge, J. Jovicevic, X. Ju, A. Juste Rozas, M. K. Köhler, A. Kaczmarska, M. Kado, H. Kagan, M. Kagan, S. J. Kahn, E. Kajomovitz, C. W. Kalderon, A. Kaluza, S. Kama, A. Kamenshchikov, N. Kanaya, S. Kaneti, L. Kanjir, V. A. Kantserov, J. Kanzaki, B. Kaplan, L. S. Kaplan, A. Kapliy, D. Kar, K. Karakostas, A. Karamaoun, N. Karastathis, M. J. Kareem, E. Karentzos, M. Karnevskiy, S. N. Karpov, Z. M. Karpova, K. Karthik, V. Kartvelishvili, A. N. Karyukhin, K. Kasahara, L. Kashif, R. D. Kass, A. Kastanas, Y. Kataoka, C. Kato, A. Katre, J. Katzy, K. Kawagoe, T. Kawamoto, G. Kawamura, S. Kazama, V. F. Kazanin, R. Keeler, R. Kehoe, J. S. Keller, J. J. Kempster, K Kentaro, H. Keoshkerian, O. Kepka, B. P. Kerševan, S. Kersten, R. A. Keyes, F. Khalil-zada, A. Khanov, A. G. Kharlamov, T. J. Khoo, V. Khovanskiy, E. Khramov, J. Khubua, S. Kido, H. Y. Kim, S. H. Kim, Y. K. Kim, N. Kimura, O. M. Kind, B. T. King, M. King, S. B. King, J. Kirk, A. E. Kiryunin, T. Kishimoto, D. Kisielewska, F. Kiss, K. Kiuchi, O. Kivernyk, E. Kladiva, M. H. Klein, M. Klein, U. Klein, K. Kleinknecht, P. Klimek, A. Klimentov, R. Klingenberg, J. A. Klinger, T. Klioutchnikova, E. -E. Kluge, P. Kluit, S. Kluth, J. Knapik, E. Kneringer, E. B. F. G. Knoops, A. Knue, A. Kobayashi, D. Kobayashi, T. Kobayashi, M. Kobel, M. Kocian, P. Kodys, T. Koffas, E. Koffeman, T. Koi, H. Kolanoski, M. Kolb, I. Koletsou, A. A. Komar, Y. Komori, T. Kondo, N. Kondrashova, K. Köneke, A. C. König, T. Kono, R. Konoplich, N. Konstantinidis, R. Kopeliansky, S. Koperny, L. Köpke, A. K. Kopp, K. Korcyl, K. Kordas, A. Korn, A. A. Korol, I. Korolkov, E. V. Korolkova, O. Kortner, S. Kortner, T. Kosek, V. V. Kostyukhin, A. Kotwal, A. Kourkoumeli-Charalampidi, C. Kourkoumelis, V. Kouskoura, A. B. Kowalewska, R. Kowalewski, T. Z. Kowalski, C. Kozakai, W. Kozanecki, A. S. Kozhin, V. A. Kramarenko, G. Kramberger, D. Krasnopevtsev, M. W. Krasny, A. Krasznahorkay, J. K. Kraus, A. Kravchenko, M. Kretz, J. Kretzschmar, K. Kreutzfeldt, P. Krieger, K. Krizka, K. Kroeninger, H. Kroha, J. Kroll, J. Kroseberg, J. Krstic, U. Kruchonak, H. Krüger, N. Krumnack, A. Kruse, M. C. Kruse, M. Kruskal, T. Kubota, H. Kucuk, S. Kuday, J. T. Kuechler, S. Kuehn, A. Kugel, F. Kuger, A. Kuhl, T. Kuhl, V. Kukhtin, R. Kukla, Y. Kulchitsky, S. Kuleshov, M. Kuna, T. Kunigo, A. Kupco, H. Kurashige, Y. A. Kurochkin, V. Kus, E. S. Kuwertz, M. Kuze, J. Kvita, T. Kwan, D. Kyriazopoulos, A. La Rosa, J. L. La Rosa Navarro, L. La Rotonda, C. Lacasta, F. Lacava, J. Lacey, H. Lacker, D. Lacour, V. R. Lacuesta, E. Ladygin, R. Lafaye, B. Laforge, T. Lagouri, S. Lai, S. Lammers, W. Lampl, E. Lançon, U. Landgraf, M. P. J. Landon, V. S. Lang, J. C. Lange, A. J. Lankford, F. Lanni, K. Lantzsch, A. Lanza, S. Laplace, C. Lapoire, J. F. Laporte, T. Lari, F. Lasagni Manghi, M. Lassnig, P. Laurelli, W. Lavrijsen, A. T. Law, P. Laycock, T. Lazovich, M. Lazzaroni, B. Le, O. Le Dortz, E. Le Guirriec, E. P. Le Quilleuc, M. LeBlanc, T. LeCompte, F. Ledroit-Guillon, C. A. Lee, S. C. Lee, L. Lee, G. Lefebvre, M. Lefebvre, F. Legger, C. Leggett, A. Lehan, G. Lehmann Miotto, X. Lei, W. A. Leight, A. Leisos, A. G. Leister, M. A. L. Leite, R. Leitner, D. Lellouch, B. Lemmer, K. J. C. Leney, T. Lenz, B. Lenzi, R. Leone, S. Leone, C. Leonidopoulos, S. Leontsinis, G. Lerner, C. Leroy, A. A. J. Lesage, C. G. Lester, M. Levchenko, J. Levêque, D. Levin, L. J. Levinson, M. Levy, D. Lewis, A. M. Leyko, M. Leyton, B. Li, H. Li, H. L. Li, L. Li, L. Li, Q. Li, S. Li, X. Li, Y. Li, Z. Liang, B. Liberti, A. Liblong, P. Lichard, K. Lie, J. Liebal, W. Liebig, A. Limosani, S. C. Lin, T. H. Lin, B. E. Lindquist, A. E. Lionti, E. Lipeles, A. Lipniacka, M. Lisovyi, T. M. Liss, A. Lister, A. M. Litke, B. Liu, D. Liu, H. Liu, H. Liu, J. Liu, J. B. Liu, K. Liu, L. Liu, M. Liu, M. Liu, Y. L. Liu, Y. Liu, M. Livan, A. Lleres, J. Llorente Merino, S. L. Lloyd, F. Lo Sterzo, E. Lobodzinska, P. Loch, W. S. Lockman, F. K. Loebinger, A. E. Loevschall-Jensen, K. M. Loew, A. Loginov, T. Lohse, K. Lohwasser, M. Lokajicek, B. A. Long, J. D. Long, R. E. Long, L. Longo, K. A. Looper, L. Lopes, D. Lopez Mateos, B. Lopez Paredes, I. Lopez Paz, A. Lopez Solis, J. Lorenz, N. Lorenzo Martinez, M. Losada, P. J. Lösel, X. Lou, A. Lounis, J. Love, P. A. Love, H. Lu, N. Lu, H. J. Lubatti, C. Luci, A. Lucotte, C. Luedtke, F. Luehring, W. Lukas, L. Luminari, O. Lundberg, B. Lund-Jensen, P. M. Luzi, D. Lynn, R. Lysak, E. Lytken, V. Lyubushkin, H. Ma, L. L. Ma, Y. Ma, G. Maccarrone, A. Macchiolo, C. M. Macdonald, B. Maček, J. Machado Miguens, D. Madaffari, R. Madar, H. J. Maddocks, W. F. Mader, A. Madsen, J. Maeda, S. Maeland, T. Maeno, A. Maevskiy, E. Magradze, J. Mahlstedt, C. Maiani, C. Maidantchik, A. A. Maier, T. Maier, A. Maio, S. Majewski, Y. Makida, N. Makovec, B. Malaescu, Pa. Malecki, V. P. Maleev, F. Malek, U. Mallik, D. Malon, C. Malone, S. Maltezos, S. Malyukov, J. Mamuzic, G. Mancini, B. Mandelli, L. Mandelli, I. Mandić, J. Maneira, L. Manhaes de Andrade Filho, J. Manjarres Ramos, A. Mann, A. Manousos, B. Mansoulie, J. D. Mansour, R. Mantifel, M. Mantoani, S. Manzoni, L. Mapelli, G. Marceca, L. March, G. Marchiori, M. Marcisovsky, M. Marjanovic, D. E. Marley, F. Marroquim, S. P. Marsden, Z. Marshall, S. Marti-Garcia, B. Martin, T. A. Martin, V. J. Martin, B. Martin dit Latour, M. Martinez, S. Martin-Haugh, V. S. Martoiu, A. C. Martyniuk, M. Marx, A. Marzin, L. Masetti, T. Mashimo, R. Mashinistov, J. Masik, A. L. Maslennikov, I. Massa, L. Massa, P. Mastrandrea, A. Mastroberardino, T. Masubuchi, P. Mättig, J. Mattmann, J. Maurer, S. J. Maxfield, D. A. Maximov, R. Mazini, S. M. Mazza, N. C. Mc Fadden, G. Mc Goldrick, S. P. Mc Kee, A. McCarn, R. L. McCarthy, T. G. McCarthy, L. I. McClymont, E. F. McDonald, K. W. McFarlane, J. A. Mcfayden, G. Mchedlidze, S. J. McMahon, R. A. McPherson, M. Medinnis, S. Meehan, S. Mehlhase, A. Mehta, K. Meier, C. Meineck, B. Meirose, D. Melini, B. R. Mellado Garcia, M. Melo, F. Meloni, A. Mengarelli, S. Menke, E. Meoni, S. Mergelmeyer, P. Mermod, L. Merola, C. Meroni, F. S. Merritt, A. Messina, J. Metcalfe, A. S. Mete, C. Meyer, C. Meyer, J-P. Meyer, J. Meyer, H. Meyer Zu Theenhausen, F. Miano, R. P. Middleton, S. Miglioranzi, L. Mijović, G. Mikenberg, M. Mikestikova, M. Mikuž, M. Milesi, A. Milic, D. W. Miller, C. Mills, A. Milov, D. A. Milstead, A. A. Minaenko, Y. Minami, I. A. Minashvili, A. I. Mincer, B. Mindur, M. Mineev, Y. Ming, L. M. Mir, K. P. Mistry, T. Mitani, J. Mitrevski, V. A. Mitsou, A. Miucci, P. S. Miyagawa, J. U. Mjörnmark, T. Moa, K. Mochizuki, S. Mohapatra, S. Molander, R. Moles-Valls, R. Monden, M. C. Mondragon, K. Mönig, J. Monk, E. Monnier, A. Montalbano, J. Montejo Berlingen, F. Monticelli, S. Monzani, R. W. Moore, N. Morange, D. Moreno, M. Moreno Llácer, P. Morettini, D. Mori, T. Mori, M. Morii, M. Morinaga, V. Morisbak, S. Moritz, A. K. Morley, G. Mornacchi, J. D. Morris, S. S. Mortensen, L. Morvaj, M. Mosidze, J. Moss, K. Motohashi, R. Mount, E. Mountricha, S. V. Mouraviev, E. J. W. Moyse, S. Muanza, R. D. Mudd, F. Mueller, J. Mueller, R. S. P. Mueller, T. Mueller, D. Muenstermann, P. Mullen, G. A. Mullier, F. J. Munoz Sanchez, J. A. Murillo Quijada, W. J. Murray, H. Musheghyan, M. Muškinja, A. G. Myagkov, M. Myska, B. P. Nachman, O. Nackenhorst, K. Nagai, R. Nagai, K. Nagano, Y. Nagasaka, K. Nagata, M. Nagel, E. Nagy, A. M. Nairz, Y. Nakahama, K. Nakamura, T. Nakamura, I. Nakano, H. Namasivayam, R. F. Naranjo Garcia, R. Narayan, D. I. Narrias Villar, I. Naryshkin, T. Naumann, G. Navarro, R. Nayyar, H. A. Neal, P. Yu. Nechaeva, T. J. Neep, P. D. Nef, A. Negri, M. Negrini, S. Nektarijevic, C. Nellist, A. Nelson, S. Nemecek, P. Nemethy, A. A. Nepomuceno, M. Nessi, M. S. Neubauer, M. Neumann, R. M. Neves, P. Nevski, P. R. Newman, D. H. Nguyen, T. Nguyen Manh, R. B. Nickerson, R. Nicolaidou, J. Nielsen, A. Nikiforov, V. Nikolaenko, I. Nikolic-Audit, K. Nikolopoulos, J. K. Nilsen, P. Nilsson, Y. Ninomiya, A. Nisati, R. Nisius, T. Nobe, L. Nodulman, M. Nomachi, I. Nomidis, T. Nooney, S. Norberg, M. Nordberg, N. Norjoharuddeen, O. Novgorodova, S. Nowak, M. Nozaki, L. Nozka, K. Ntekas, E. Nurse, F. Nuti, F. O’grady, D. C. O’Neil, A. A. O’Rourke, V. O’Shea, F. G. Oakham, H. Oberlack, T. Obermann, J. Ocariz, A. Ochi, I. Ochoa, J. P. Ochoa-Ricoux, S. Oda, S. Odaka, H. Ogren, A. Oh, S. H. Oh, C. C. Ohm, H. Ohman, H. Oide, H. Okawa, Y. Okumura, T. Okuyama, A. Olariu, L. F. Oleiro Seabra, S. A. Olivares Pino, D. Oliveira Damazio, A. Olszewski, J. Olszowska, A. Onofre, K. Onogi, P. U. E. Onyisi, M. J. Oreglia, Y. Oren, D. Orestano, N. Orlando, R. S. Orr, B. Osculati, R. Ospanov, G. Otero y Garzon, H. Otono, M. Ouchrif, F. Ould-Saada, A. Ouraou, K. P. Oussoren, Q. Ouyang, M. Owen, R. E. Owen, V. E. Ozcan, N. Ozturk, K. Pachal, A. Pacheco Pages, C. Padilla Aranda, M. Pagáčová, S. Pagan Griso, F. Paige, P. Pais, K. Pajchel, G. Palacino, S. Palestini, M. Palka, D. Pallin, A. Palma, E. St. Panagiotopoulou, C. E. Pandini, J. G. Panduro Vazquez, P. Pani, S. Panitkin, D. Pantea, L. Paolozzi, Th. D. Papadopoulou, K. Papageorgiou, A. Paramonov, D. Paredes Hernandez, A. J. Parker, M. A. Parker, K. A. Parker, F. Parodi, J. A. Parsons, U. Parzefall, V. R. Pascuzzi, E. Pasqualucci, S. Passaggio, Fr. Pastore, G. Pásztor, S. Pataraia, J. R. Pater, T. Pauly, J. Pearce, B. Pearson, L. E. Pedersen, M. Pedersen, S. Pedraza Lopez, R. Pedro, S. V. Peleganchuk, D. Pelikan, O. Penc, C. Peng, H. Peng, J. Penwell, B. S. Peralva, M. M. Perego, D. V. Perepelitsa, E. Perez Codina, L. Perini, H. Pernegger, S. Perrella, R. Peschke, V. D. Peshekhonov, K. Peters, R. F. Y. Peters, B. A. Petersen, T. C. Petersen, E. Petit, A. Petridis, C. Petridou, P. Petroff, E. Petrolo, M. Petrov, F. Petrucci, N. E. Pettersson, A. Peyaud, R. Pezoa, P. W. Phillips, G. Piacquadio, E. Pianori, A. Picazio, E. Piccaro, M. Piccinini, M. A. Pickering, R. Piegaia, J. E. Pilcher, A. D. Pilkington, A. W. J. Pin, M. Pinamonti, J. L. Pinfold, A. Pingel, S. Pires, H. Pirumov, M. Pitt, L. Plazak, M. -A. Pleier, V. Pleskot, E. Plotnikova, P. Plucinski, D. Pluth, R. Poettgen, L. Poggioli, D. Pohl, G. Polesello, A. Poley, A. Policicchio, R. Polifka, A. Polini, C. S. Pollard, V. Polychronakos, K. Pommès, L. Pontecorvo, B. G. Pope, G. A. Popeneciu, D. S. Popovic, A. Poppleton, S. Pospisil, K. Potamianos, I. N. Potrap, C. J. Potter, C. T. Potter, G. Poulard, J. Poveda, V. Pozdnyakov, M. E. Pozo Astigarraga, P. Pralavorio, A. Pranko, S. Prell, D. Price, L. E. Price, M. Primavera, S. Prince, M. Proissl, K. Prokofiev, F. Prokoshin, S. Protopopescu, J. Proudfoot, M. Przybycien, D. Puddu, D. Puldon, M. Purohit, P. Puzo, J. Qian, G. Qin, Y. Qin, A. Quadt, W. B. Quayle, M. Queitsch-Maitland, D. Quilty, S. Raddum, V. Radeka, V. Radescu, S. K. Radhakrishnan, P. Radloff, P. Rados, F. Ragusa, G. Rahal, J. A. Raine, S. Rajagopalan, M. Rammensee, C. Rangel-Smith, M. G. Ratti, F. Rauscher, S. Rave, T. Ravenscroft, I. Ravinovich, M. Raymond, A. L. Read, N. P. Readioff, M. Reale, D. M. Rebuzzi, A. Redelbach, G. Redlinger, R. Reece, K. Reeves, L. Rehnisch, J. Reichert, H. Reisin, C. Rembser, H. Ren, M. Rescigno, S. Resconi, O. L. Rezanova, P. Reznicek, R. Rezvani, R. Richter, S. Richter, E. Richter-Was, O. Ricken, M. Ridel, P. Rieck, C. J. Riegel, J. Rieger, O. Rifki, M. Rijssenbeek, A. Rimoldi, M. Rimoldi, L. Rinaldi, B. Ristić, E. Ritsch, I. Riu, F. Rizatdinova, E. Rizvi, C. Rizzi, S. H. Robertson, A. Robichaud-Veronneau, D. Robinson, J. E. M. Robinson, A. Robson, C. Roda, Y. Rodina, A. Rodriguez Perez, D. Rodriguez Rodriguez, S. Roe, C. S. Rogan, O. Røhne, A. Romaniouk, M. Romano, S. M. Romano Saez, E. Romero Adam, N. Rompotis, M. Ronzani, L. Roos, E. Ros, S. Rosati, K. Rosbach, P. Rose, O. Rosenthal, N. -A. Rosien, V. Rossetti, E. Rossi, L. P. Rossi, J. H. N. Rosten, R. Rosten, M. Rotaru, I. Roth, J. Rothberg, D. Rousseau, C. R. Royon, A. Rozanov, Y. Rozen, X. Ruan, F. Rubbo, M. S. Rudolph, F. Rühr, A. Ruiz-Martinez, Z. Rurikova, N. A. Rusakovich, A. Ruschke, H. L. Russell, J. P. Rutherfoord, N. Ruthmann, Y. F. Ryabov, M. Rybar, G. Rybkin, S. Ryu, A. Ryzhov, G. F. Rzehorz, A. F. Saavedra, G. Sabato, S. Sacerdoti, H. F-W. Sadrozinski, R. Sadykov, F. Safai Tehrani, P. Saha, M. Sahinsoy, M. Saimpert, T. Saito, H. Sakamoto, Y. Sakurai, G. Salamanna, A. Salamon, J. E. Salazar Loyola, D. Salek, P. H. Sales De Bruin, D. Salihagic, A. Salnikov, J. Salt, D. Salvatore, F. Salvatore, A. Salvucci, A. Salzburger, D. Sammel, D. Sampsonidis, A. Sanchez, J. Sánchez, V. Sanchez Martinez, H. Sandaker, R. L. Sandbach, H. G. Sander, M. Sandhoff, C. Sandoval, R. Sandstroem, D. P. C. Sankey, M. Sannino, A. Sansoni, C. Santoni, R. Santonico, H. Santos, I. Santoyo Castillo, K. Sapp, A. Sapronov, J. G. Saraiva, B. Sarrazin, O. Sasaki, Y. Sasaki, K. Sato, G. Sauvage, E. Sauvan, G. Savage, P. Savard, C. Sawyer, L. Sawyer, J. Saxon, C. Sbarra, A. Sbrizzi, T. Scanlon, D. A. Scannicchio, M. Scarcella, V. Scarfone, J. Schaarschmidt, P. Schacht, B. M. Schachtner, D. Schaefer, R. Schaefer, J. Schaeffer, S. Schaepe, S. Schaetzel, U. Schäfer, A. C. Schaffer, D. Schaile, R. D. Schamberger, V. Scharf, V. A. Schegelsky, D. Scheirich, M. Schernau, C. Schiavi, S. Schier, C. Schillo, M. Schioppa, S. Schlenker, K. R. Schmidt-Sommerfeld, K. Schmieden, C. Schmitt, S. Schmitt, S. Schmitz, B. Schneider, U. Schnoor, L. Schoeffel, A. Schoening, B. D. Schoenrock, E. Schopf, M. Schott, J. Schovancova, S. Schramm, M. Schreyer, N. Schuh, M. J. Schultens, H. -C. Schultz-Coulon, H. Schulz, M. Schumacher, B. A. Schumm, Ph. Schune, A. Schwartzman, T. A. Schwarz, Ph. Schwegler, H. Schweiger, Ph. Schwemling, R. Schwienhorst, J. Schwindling, T. Schwindt, G. Sciolla, F. Scuri, F. Scutti, J. Searcy, P. Seema, S. C. Seidel, A. Seiden, F. Seifert, J. M. Seixas, G. Sekhniaidze, K. Sekhon, S. J. Sekula, D. M. Seliverstov, N. Semprini-Cesari, C. Serfon, L. Serin, L. Serkin, M. Sessa, R. Seuster, H. Severini, T. Sfiligoj, F. Sforza, A. Sfyrla, E. Shabalina, N. W. Shaikh, L. Y. Shan, R. Shang, J. T. Shank, M. Shapiro, P. B. Shatalov, K. Shaw, S. M. Shaw, A. Shcherbakova, C. Y. Shehu, P. Sherwood, L. Shi, S. Shimizu, C. O. Shimmin, M. Shimojima, M. Shiyakova, A. Shmeleva, D. Shoaleh Saadi, M. J. Shochet, S. Shojaii, S. Shrestha, E. Shulga, M. A. Shupe, P. Sicho, A. M. Sickles, P. E. Sidebo, O. Sidiropoulou, D. Sidorov, A. Sidoti, F. Siegert, Dj. Sijacki, J. Silva, S. B. Silverstein, V. Simak, O. Simard, Lj. Simic, S. Simion, E. Simioni, B. Simmons, D. Simon, M. Simon, P. Sinervo, N. B. Sinev, M. Sioli, G. Siragusa, S. Yu. Sivoklokov, J. Sjölin, T. B. Sjursen, M. B. Skinner, H. P. Skottowe, P. Skubic, M. Slater, T. Slavicek, M. Slawinska, K. Sliwa, R. Slovak, V. Smakhtin, B. H. Smart, L. Smestad, J. Smiesko, S. Yu. Smirnov, Y. Smirnov, L. N. Smirnova, O. Smirnova, M. N. K. Smith, R. W. Smith, M. Smizanska, K. Smolek, A. A. Snesarev, S. Snyder, R. Sobie, F. Socher, A. Soffer, D. A. Soh, G. Sokhrannyi, C. A. Solans Sanchez, M. Solar, E. Yu. Soldatov, U. Soldevila, A. A. Solodkov, A. Soloshenko, O. V. Solovyanov, V. Solovyev, P. Sommer, H. Son, H. Y. Song, A. Sood, A. Sopczak, V. Sopko, V. Sorin, D. Sosa, C. L. Sotiropoulou, R. Soualah, A. M. Soukharev, D. South, B. C. Sowden, S. Spagnolo, M. Spalla, M. Spangenberg, F. Spanò, D. Sperlich, F. Spettel, R. Spighi, G. Spigo, L. A. Spiller, M. Spousta, R. D. St. Denis, A. Stabile, R. Stamen, S. Stamm, E. Stanecka, R. W. Stanek, C. Stanescu, M. Stanescu-Bellu, M. M. Stanitzki, S. Stapnes, E. A. Starchenko, G. H. Stark, J. Stark, P. Staroba, P. Starovoitov, S. Stärz, R. Staszewski, P. Steinberg, B. Stelzer, H. J. Stelzer, O. Stelzer-Chilton, H. Stenzel, G. A. Stewart, J. A. Stillings, M. C. Stockton, M. Stoebe, G. Stoicea, P. Stolte, S. Stonjek, A. R. Stradling, A. Straessner, M. E. Stramaglia, J. Strandberg, S. Strandberg, A. Strandlie, M. Strauss, P. Strizenec, R. Ströhmer, D. M. Strom, R. Stroynowski, A. Strubig, S. A. Stucci, B. Stugu, N. A. Styles, D. Su, J. Su, R. Subramaniam, S. Suchek, Y. Sugaya, M. Suk, V. V. Sulin, S. Sultansoy, T. Sumida, S. Sun, X. Sun, J. E. Sundermann, K. Suruliz, G. Susinno, M. R. Sutton, S. Suzuki, M. Svatos, M. Swiatlowski, I. Sykora, T. Sykora, D. Ta, C. Taccini, K. Tackmann, J. Taenzer, A. Taffard, R. Tafirout, N. Taiblum, H. Takai, R. Takashima, T. Takeshita, Y. Takubo, M. Talby, A. A. Talyshev, K. G. Tan, J. Tanaka, R. Tanaka, S. Tanaka, B. B. Tannenwald, S. Tapia Araya, S. Tapprogge, S. Tarem, G. F. Tartarelli, P. Tas, M. Tasevsky, T. Tashiro, E. Tassi, A. Tavares Delgado, Y. Tayalati, A. C. Taylor, G. N. Taylor, P. T. E. Taylor, W. Taylor, F. A. Teischinger, P. Teixeira-Dias, K. K. Temming, D. Temple, H. Ten Kate, P. K. Teng, J. J. Teoh, F. Tepel, S. Terada, K. Terashi, J. Terron, S. Terzo, M. Testa, R. J. Teuscher, T. Theveneaux-Pelzer, J. P. Thomas, J. Thomas-Wilsker, E. N. Thompson, P. D. Thompson, A. S. Thompson, L. A. Thomsen, E. Thomson, M. Thomson, M. J. Tibbetts, R. E. Ticse Torres, V. O. Tikhomirov, Yu. A. Tikhonov, S. Timoshenko, P. Tipton, S. Tisserant, K. Todome, T. Todorov, S. Todorova-Nova, J. Tojo, S. Tokár, K. Tokushuku, E. Tolley, L. Tomlinson, M. Tomoto, L. Tompkins, K. Toms, B. Tong, E. Torrence, H. Torres, E. Torró Pastor, J. Toth, F. Touchard, D. R. Tovey, T. Trefzger, F. Tresoldi, A. Tricoli, I. M. Trigger, S. Trincaz-Duvoid, M. F. Tripiana, W. Trischuk, B. Trocmé, A. Trofymov, C. Troncon, M. Trottier-McDonald, M. Trovatelli, L. Truong, M. Trzebinski, A. Trzupek, J. C-L. Tseng, P. V. Tsiareshka, G. Tsipolitis, N. Tsirintanis, S. Tsiskaridze, V. Tsiskaridze, E. G. Tskhadadze, K. M. Tsui, I. I. Tsukerman, V. Tsulaia, S. Tsuno, D. Tsybychev, A. Tudorache, V. Tudorache, A. N. Tuna, S. A. Tupputi, S. Turchikhin, D. Turecek, D. Turgeman, R. Turra, A. J. Turvey, P. M. Tuts, M. Tyndel, G. Ucchielli, I. Ueda, R. Ueno, M. Ughetto, F. Ukegawa, G. Unal, A. Undrus, G. Unel, F. C. Ungaro, Y. Unno, C. Unverdorben, J. Urban, P. Urquijo, P. Urrejola, G. Usai, A. Usanova, L. Vacavant, V. Vacek, B. Vachon, C. Valderanis, E. Valdes Santurio, N. Valencic, S. Valentinetti, A. Valero, L. Valery, S. Valkar, S. Vallecorsa, J. A. Valls Ferrer, W. Van Den Wollenberg, P. C. Van Der Deijl, R. van der Geer, H. van der Graaf, N. van Eldik, P. van Gemmeren, J. Van Nieuwkoop, I. van Vulpen, M. C. van Woerden, M. Vanadia, W. Vandelli, R. Vanguri, A. Vaniachine, P. Vankov, G. Vardanyan, R. Vari, E. W. Varnes, T. Varol, D. Varouchas, A. Vartapetian, K. E. Varvell, J. G. Vasquez, F. Vazeille, T. Vazquez Schroeder, J. Veatch, L. M. Veloce, F. Veloso, S. Veneziano, A. Ventura, M. Venturi, N. Venturi, A. Venturini, V. Vercesi, M. Verducci, W. Verkerke, J. C. Vermeulen, A. Vest, M. C. Vetterli, O. Viazlo, I. Vichou, T. Vickey, O. E. Vickey Boeriu, G. H. A. Viehhauser, S. Viel, L. Vigani, R. Vigne, M. Villa, M. Villaplana Perez, E. Vilucchi, M. G. Vincter, V. B. Vinogradov, C. Vittori, I. Vivarelli, S. Vlachos, M. Vlasak, M. Vogel, P. Vokac, G. Volpi, M. Volpi, H. von der Schmitt, E. von Toerne, V. Vorobel, K. Vorobev, M. Vos, R. Voss, J. H. Vossebeld, N. Vranjes, M. Vranjes Milosavljevic, V. Vrba, M. Vreeswijk, R. Vuillermet, I. Vukotic, Z. Vykydal, P. Wagner, W. Wagner, H. Wahlberg, S. Wahrmund, J. Wakabayashi, J. Walder, R. Walker, W. Walkowiak, V. Wallangen, C. Wang, C. Wang, F. Wang, H. Wang, H. Wang, J. Wang, J. Wang, K. Wang, R. Wang, S. M. Wang, T. Wang, T. Wang, W. Wang, X. Wang, C. Wanotayaroj, A. Warburton, C. P. Ward, D. R. Wardrope, A. Washbrook, P. M. Watkins, A. T. Watson, M. F. Watson, G. Watts, S. Watts, B. M. Waugh, S. Webb, M. S. Weber, S. W. Weber, J. S. Webster, A. R. Weidberg, B. Weinert, J. Weingarten, C. Weiser, H. Weits, P. S. Wells, T. Wenaus, T. Wengler, S. Wenig, N. Wermes, M. Werner, P. Werner, M. Wessels, J. Wetter, K. Whalen, N. L. Whallon, A. M. Wharton, A. White, M. J. White, R. White, D. Whiteson, F. J. Wickens, W. Wiedenmann, M. Wielers, P. Wienemann, C. Wiglesworth, L. A. M. Wiik-Fuchs, A. Wildauer, F. Wilk, H. G. Wilkens, H. H. Williams, S. Williams, C. Willis, S. Willocq, J. A. Wilson, I. Wingerter-Seez, F. Winklmeier, O. J. Winston, B. T. Winter, M. Wittgen, J. Wittkowski, S. J. Wollstadt, M. W. Wolter, H. Wolters, B. K. Wosiek, J. Wotschack, M. J. Woudstra, K. W. Wozniak, M. Wu, M. Wu, S. L. Wu, X. Wu, Y. Wu, T. R. Wyatt, B. M. Wynne, S. Xella, D. Xu, L. Xu, B. Yabsley, S. Yacoob, R. Yakabe, D. Yamaguchi, Y. Yamaguchi, A. Yamamoto, S. Yamamoto, T. Yamanaka, K. Yamauchi, Y. Yamazaki, Z. Yan, H. Yang, H. Yang, Y. Yang, Z. Yang, W-M. Yao, Y. C. Yap, Y. Yasu, E. Yatsenko, K. H. Yau Wong, J. Ye, S. Ye, I. Yeletskikh, A. L. Yen, E. Yildirim, K. Yorita, R. Yoshida, K. Yoshihara, C. Young, C. J. S. Young, S. Youssef, D. R. Yu, J. Yu, J. M. Yu, J. Yu, L. Yuan, S. P. Y. Yuen, I. Yusuff, B. Zabinski, R. Zaidan, A. M. Zaitsev, N. Zakharchuk, J. Zalieckas, A. Zaman, S. Zambito, L. Zanello, D. Zanzi, C. Zeitnitz, M. Zeman, A. Zemla, J. C. Zeng, Q. Zeng, K. Zengel, O. Zenin, T. Ženiš, D. Zerwas, D. Zhang, F. Zhang, G. Zhang, H. Zhang, J. Zhang, L. Zhang, R. Zhang, R. Zhang, X. Zhang, Z. Zhang, X. Zhao, Y. Zhao, Z. Zhao, A. Zhemchugov, J. Zhong, B. Zhou, C. Zhou, L. Zhou, L. Zhou, M. Zhou, N. Zhou, C. G. Zhu, H. Zhu, J. Zhu, Y. Zhu, X. Zhuang, K. Zhukov, A. Zibell, D. Zieminska, N. I. Zimine, C. Zimmermann, S. Zimmermann, Z. Zinonos, M. Zinser, M. Ziolkowski, L. Živković, G. Zobernig, A. Zoccoli, M. zur Nedden, G. Zurzolo, L. Zwalinski, ATLAS Collaboration

**Affiliations:** 1 Department of Physics, University of Adelaide, Adelaide, Australia; 2 Physics Department, SUNY Albany, Albany, NY USA; 3 Department of Physics, University of Alberta, Edmonton, AB Canada; 4 Department of Physics, Ankara University, Ankara, Turkey; 5 Istanbul Aydin University, Istanbul, Turkey; 6 Division of Physics, TOBB University of Economics and Technology, Ankara, Turkey; 7 LAPP, CNRS/IN2P3 and Université Savoie Mont Blanc, Annecy-le-Vieux, France; 8 High Energy Physics Division, Argonne National Laboratory, Argonne, IL USA; 9 Department of Physics, University of Arizona, Tucson, AZ USA; 10 Department of Physics, The University of Texas at Arlington, Arlington, TX USA; 11 Physics Department, University of Athens, Athens, Greece; 12 Physics Department, National Technical University of Athens, Zografou, Greece; 13 Department of Physics, The University of Texas at Austin, Austin, TX USA; 14 Institute of Physics, Azerbaijan Academy of Sciences, Baku, Azerbaijan; 15 Institut de Física d’Altes Energies (IFAE), The Barcelona Institute of Science and Technology, Barcelona, Spain; 16 Institute of Physics, University of Belgrade, Belgrade, Serbia; 17 Department for Physics and Technology, University of Bergen, Bergen, Norway; 18 Physics Division, Lawrence Berkeley National Laboratory and University of California, Berkeley, CA USA; 19 Department of Physics, Humboldt University, Berlin, Germany; 20 Albert Einstein Center for Fundamental Physics and Laboratory for High Energy Physics, University of Bern, Bern, Switzerland; 21 School of Physics and Astronomy, University of Birmingham, Birmingham, UK; 22 Department of Physics, Bogazici University, Istanbul, Turkey; 23 Department of Physics Engineering, Gaziantep University, Gaziantep, Turkey; 24 Faculty of Engineering and Natural Sciences, Istanbul Bilgi University, Istanbul, Turkey; 25 Faculty of Engineering and Natural Sciences, Bahcesehir University, Istanbul, Turkey; 26 Centro de Investigaciones, Universidad Antonio Narino, Bogota, Colombia; 27 INFN Sezione di Bologna, Bologna, Italy; 28 Dipartimento di Fisica e Astronomia, Università di Bologna, Bologna, Italy; 29 Physikalisches Institut, University of Bonn, Bonn, Germany; 30 Department of Physics, Boston University, Boston, MA USA; 31 Department of Physics, Brandeis University, Waltham, MA USA; 32 Universidade Federal do Rio De Janeiro COPPE/EE/IF, Rio de Janeiro, Brazil; 33 Electrical Circuits Department, Federal University of Juiz de Fora (UFJF), Juiz de Fora, Brazil; 34 Federal University of Sao Joao del Rei (UFSJ), Sao Joao del Rei, Brazil; 35 Instituto de Fisica, Universidade de Sao Paulo, São Paulo, Brazil; 36 Physics Department, Brookhaven National Laboratory, Upton, NY USA; 37 Transilvania University of Brasov, Brasov, Romania; 38 National Institute of Physics and Nuclear Engineering, Bucharest, Romania; 39 Physics Department, National Institute for Research and Development of Isotopic and Molecular Technologies, Cluj Napoca, Romania; 40 University Politehnica Bucharest, Bucharest, Romania; 41 West University in Timisoara, Timisoara, Romania; 42 Departamento de Física, Universidad de Buenos Aires, Buenos Aires, Argentina; 43 Cavendish Laboratory, University of Cambridge, Cambridge, UK; 44 Department of Physics, Carleton University, Ottawa, ON Canada; 45 CERN, Geneva, Switzerland; 46 Enrico Fermi Institute, University of Chicago, Chicago, IL USA; 47 Departamento de Física, Pontificia Universidad Católica de Chile, Santiago, Chile; 48 Departamento de Física, Universidad Técnica Federico Santa María, Valparaiso, Chile; 49 Institute of High Energy Physics, Chinese Academy of Sciences, Beijing, China; 50 Department of Modern Physics, University of Science and Technology of China, Hefei, Anhui China; 51 Department of Physics, Nanjing University, Nanjing, Jiangsu China; 52 School of Physics, Shandong University, Jinan, Shandong China; 53 Shanghai Key Laboratory for Particle Physics and Cosmology, Department of Physics and Astronomy, Shanghai Jiao Tong University; (also affiliated with PKU-CHEP), Shanghai, China; 54 Physics Department, Tsinghua University, Beijing, 100084 China; 55 Laboratoire de Physique Corpusculaire, Clermont Université and Université Blaise Pascal and CNRS/IN2P3, Clermont-Ferrand, France; 56 Nevis Laboratory, Columbia University, Irvington, NY USA; 57 Niels Bohr Institute, University of Copenhagen, Kobenhavn, Denmark; 58 INFN Gruppo Collegato di Cosenza, Laboratori Nazionali di Frascati, Frascati, Italy; 59 Dipartimento di Fisica, Università della Calabria, Rende, Italy; 60 Faculty of Physics and Applied Computer Science, AGH University of Science and Technology, Kraków, Poland; 61 Marian Smoluchowski Institute of Physics, Jagiellonian University, Kraków, Poland; 62 Institute of Nuclear Physics, Polish Academy of Sciences, Kraków, Poland; 63 Physics Department, Southern Methodist University, Dallas, TX USA; 64 Physics Department, University of Texas at Dallas, Richardson, TX USA; 65 DESY, Hamburg and Zeuthen, Germany; 66 Institut für Experimentelle Physik IV, Technische Universität Dortmund, Dortmund, Germany; 67 Institut für Kern- und Teilchenphysik, Technische Universität Dresden, Dresden, Germany; 68 Department of Physics, Duke University, Durham, NC USA; 69 SUPA-School of Physics and Astronomy, University of Edinburgh, Edinburgh, UK; 70 INFN Laboratori Nazionali di Frascati, Frascati, Italy; 71 Fakultät für Mathematik und Physik, Albert-Ludwigs-Universität, Freiburg, Germany; 72 Section de Physique, Université de Genève, Geneva, Switzerland; 73 INFN Sezione di Genova, Genova, Italy; 74 Dipartimento di Fisica, Università di Genova, Genova, Italy; 75 E. Andronikashvili Institute of Physics, Iv. Javakhishvili Tbilisi State University, Tbilisi, Georgia; 76 High Energy Physics Institute, Tbilisi State University, Tbilisi, Georgia; 77 II Physikalisches Institut, Justus-Liebig-Universität Giessen, Giessen, Germany; 78 SUPA-School of Physics and Astronomy, University of Glasgow, Glasgow, UK; 79 II Physikalisches Institut, Georg-August-Universität, Göttingen, Germany; 80 Laboratoire de Physique Subatomique et de Cosmologie, Université Grenoble-Alpes, CNRS/IN2P3, Grenoble, France; 81 Department of Physics, Hampton University, Hampton, VA USA; 82 Laboratory for Particle Physics and Cosmology, Harvard University, Cambridge, MA USA; 83 Kirchhoff-Institut für Physik, Ruprecht-Karls-Universität Heidelberg, Heidelberg, Germany; 84 Physikalisches Institut, Ruprecht-Karls-Universität Heidelberg, Heidelberg, Germany; 85 ZITI Institut für technische Informatik, Ruprecht-Karls-Universität Heidelberg, Mannheim, Germany; 86 Faculty of Applied Information Science, Hiroshima Institute of Technology, Hiroshima, Japan; 87 Department of Physics, The Chinese University of Hong Kong, Shatin, NT Hong Kong; 88 Department of Physics, The University of Hong Kong, Hong Kong, China; 89 Department of Physics, The Hong Kong University of Science and Technology, Clear Water Bay, Kowloon, Hong Kong, China; 90 Department of Physics, Indiana University, Bloomington, IN USA; 91 Institut für Astro- und Teilchenphysik, Leopold-Franzens-Universität, Innsbruck, Austria; 92 University of Iowa, Iowa City, IA USA; 93 Department of Physics and Astronomy, Iowa State University, Ames, IA USA; 94 Joint Institute for Nuclear Research, JINR Dubna, Dubna, Russia; 95 KEK, High Energy Accelerator Research Organization, Tsukuba, Japan; 96 Graduate School of Science, Kobe University, Kobe, Japan; 97 Faculty of Science, Kyoto University, Kyoto, Japan; 98 Kyoto University of Education, Kyoto, Japan; 99 Department of Physics, Kyushu University, Fukuoka, Japan; 100 Instituto de Física La Plata, Universidad Nacional de La Plata and CONICET, La Plata, Argentina; 101 Physics Department, Lancaster University, Lancaster, UK; 102 INFN Sezione di Lecce, Lecce, Italy; 103 Dipartimento di Matematica e Fisica, Università del Salento, Lecce, Italy; 104 Oliver Lodge Laboratory, University of Liverpool, Liverpool, UK; 105 Department of Physics, Jožef Stefan Institute and University of Ljubljana, Ljubljana, Slovenia; 106 School of Physics and Astronomy, Queen Mary University of London, London, UK; 107 Department of Physics, Royal Holloway University of London, Surrey, UK; 108 Department of Physics and Astronomy, University College London, London, UK; 109 Louisiana Tech University, Ruston, LA USA; 110 Laboratoire de Physique Nucléaire et de Hautes Energies, UPMC and Université Paris-Diderot and CNRS/IN2P3, Paris, France; 111 Fysiska institutionen, Lunds universitet, Lund, Sweden; 112 Departamento de Fisica Teorica C-15, Universidad Autonoma de Madrid, Madrid, Spain; 113 Institut für Physik, Universität Mainz, Mainz, Germany; 114 School of Physics and Astronomy, University of Manchester, Manchester, UK; 115 CPPM, Aix-Marseille Université and CNRS/IN2P3, Marseille, France; 116 Department of Physics, University of Massachusetts, Amherst, MA USA; 117 Department of Physics, McGill University, Montreal, QC Canada; 118 School of Physics, University of Melbourne, Melbourne, VIC Australia; 119 Department of Physics, The University of Michigan, Ann Arbor, MI USA; 120 Department of Physics and Astronomy, Michigan State University, East Lansing, MI USA; 121 INFN Sezione di Milano, Milan, Italy; 122 Dipartimento di Fisica, Università di Milano, Milan, Italy; 123 B.I. Stepanov Institute of Physics, National Academy of Sciences of Belarus, Minsk, Republic of Belarus; 124 National Scientific and Educational Centre for Particle and High Energy Physics, Minsk, Republic of Belarus; 125 Group of Particle Physics, University of Montreal, Montreal, QC Canada; 126 P.N. Lebedev Physical Institute of the Russian, Academy of Sciences, Moscow, Russia; 127 Institute for Theoretical and Experimental Physics (ITEP), Moscow, Russia; 128 National Research Nuclear University MEPhI, Moscow, Russia; 129 D.V. Skobeltsyn Institute of Nuclear Physics, M.V. Lomonosov Moscow State University, Moscow, Russia; 130 Fakultät für Physik, Ludwig-Maximilians-Universität München, München, Germany; 131 Max-Planck-Institut für Physik (Werner-Heisenberg-Institut), Munich, Germany; 132 Nagasaki Institute of Applied Science, Nagasaki, Japan; 133 Graduate School of Science and Kobayashi-Maskawa Institute, Nagoya University, Nagoya, Japan; 134 INFN Sezione di Napoli, Naples, Italy; 135 Dipartimento di Fisica, Università di Napoli, Naples, Italy; 136 Department of Physics and Astronomy, University of New Mexico, Albuquerque, NM USA; 137 Institute for Mathematics, Astrophysics and Particle Physics, Radboud University Nijmegen/Nikhef, Nijmegen, The Netherlands; 138 Nikhef National Institute for Subatomic Physics and University of Amsterdam, Amsterdam, The Netherlands; 139 Department of Physics, Northern Illinois University, DeKalb, IL USA; 140 Budker Institute of Nuclear Physics, SB RAS, Novosibirsk, Russia; 141 Department of Physics, New York University, New York, NY USA; 142 Ohio State University, Columbus, OH USA; 143 Faculty of Science, Okayama University, Okayama, Japan; 144 Homer L. Dodge Department of Physics and Astronomy, University of Oklahoma, Norman, OK USA; 145 Department of Physics, Oklahoma State University, Stillwater, OK USA; 146 Palacký University, RCPTM, Olomouc, Czech Republic; 147 Center for High Energy Physics, University of Oregon, Eugene, OR USA; 148 LAL, Univ. Paris-Sud, CNRS/IN2P3, Université Paris Saclay, Orsay, France; 149 Graduate School of Science, Osaka University, Osaka, Japan; 150 Department of Physics, University of Oslo, Oslo, Norway; 151 Department of Physics, Oxford University, Oxford, UK; 152 INFN Sezione di Pavia, Pavia, Italy; 153 Dipartimento di Fisica, Università di Pavia, Pavia, Italy; 154 Department of Physics, University of Pennsylvania, Philadelphia, PA USA; 155 National Research Centre “Kurchatov Institute” B.P.Konstantinov Petersburg Nuclear Physics Institute, St. Petersburg, Russia; 156 INFN Sezione di Pisa, Pisa, Italy; 157 Dipartimento di Fisica E. Fermi, Università di Pisa, Pisa, Italy; 158 Department of Physics and Astronomy, University of Pittsburgh, Pittsburgh, PA USA; 159 Laboratório de Instrumentação e Física Experimental de Partículas-LIP, Lisboa, Portugal; 160 Faculdade de Ciências, Universidade de Lisboa, Lisbon, Portugal; 161 Department of Physics, University of Coimbra, Coimbra, Portugal; 162 Centro de Física Nuclear da Universidade de Lisboa, Lisbon, Portugal; 163 Departamento de Fisica, Universidade do Minho, Braga, Portugal; 164 Departamento de Fisica Teorica y del Cosmos and CAFPE, Universidad de Granada, Granada, Spain; 165 Dep Fisica and CEFITEC of Faculdade de Ciencias e Tecnologia, Universidade Nova de Lisboa, Caparica, Portugal; 166 Institute of Physics, Academy of Sciences of the Czech Republic, Praha, Czech Republic; 167 Czech Technical University in Prague, Praha, Czech Republic; 168 Faculty of Mathematics and Physics, Charles University in Prague, Praha, Czech Republic; 169 State Research Center Institute for High Energy Physics, (Protvino), NRC KI, Moscow, Russia; 170 Particle Physics Department, Rutherford Appleton Laboratory, Didcot, UK; 171 INFN Sezione di Roma, Rome, Italy; 172 Dipartimento di Fisica, Sapienza Università di Roma, Rome, Italy; 173 INFN Sezione di Roma Tor Vergata, Rome, Italy; 174 Dipartimento di Fisica, Università di Roma Tor Vergata, Rome, Italy; 175 INFN Sezione di Roma Tre, Rome, Italy; 176 Dipartimento di Matematica e Fisica, Università Roma Tre, Rome, Italy; 177 Faculté des Sciences Ain Chock, Réseau Universitaire de Physique des Hautes Energies-Université Hassan II, Casablanca, Morocco; 178 Centre National de l’Energie des Sciences Techniques Nucleaires, Rabat, Morocco; 179 Faculté des Sciences Semlalia, Université Cadi Ayyad, LPHEA-Marrakech, Marrakech, Morocco; 180 Faculté des Sciences, Université Mohamed Premier and LPTPM, Oujda, Morocco; 181 Faculté des Sciences, Université Mohammed V, Rabat, Morocco; 182 DSM/IRFU (Institut de Recherches sur les Lois Fondamentales de l’Univers), CEA Saclay (Commissariat à l’Energie Atomique et aux Energies Alternatives), Gif-sur-Yvette, France; 183 Santa Cruz Institute for Particle Physics, University of California Santa Cruz, Santa Cruz, CA USA; 184 Department of Physics, University of Washington, Seattle, WA USA; 185 Department of Physics and Astronomy, University of Sheffield, Sheffield, UK; 186 Department of Physics, Shinshu University, Nagano, Japan; 187 Fachbereich Physik, Universität Siegen, Siegen, Germany; 188 Department of Physics, Simon Fraser University, Burnaby, BC Canada; 189 SLAC National Accelerator Laboratory, Stanford, CA USA; 190 Faculty of Mathematics, Physics and Informatics, Comenius University, Bratislava, Slovak Republic; 191 Department of Subnuclear Physics, Institute of Experimental Physics of the Slovak Academy of Sciences, Kosice, Slovak Republic; 192 Department of Physics, University of Cape Town, Cape Town, South Africa; 193 Department of Physics, University of Johannesburg, Johannesburg, South Africa; 194 School of Physics, University of the Witwatersrand, Johannesburg, South Africa; 195 Department of Physics, Stockholm University, Stockholm, Sweden; 196 The Oskar Klein Centre, Stockholm, Sweden; 197 Physics Department, Royal Institute of Technology, Stockholm, Sweden; 198 Departments of Physics and Astronomy and Chemistry, Stony Brook University, Stony Brook, NY USA; 199 Department of Physics and Astronomy, University of Sussex, Brighton, UK; 200 School of Physics, University of Sydney, Sydney, Australia; 201 Institute of Physics, Academia Sinica, Taipei, Taiwan; 202 Department of Physics, Technion: Israel Institute of Technology, Haifa, Israel; 203 Raymond and Beverly Sackler School of Physics and Astronomy, Tel Aviv University, Tel Aviv, Israel; 204 Department of Physics, Aristotle University of Thessaloniki, Thessaloníki, Greece; 205 International Center for Elementary Particle Physics and Department of Physics, The University of Tokyo, Tokyo, Japan; 206 Graduate School of Science and Technology, Tokyo Metropolitan University, Tokyo, Japan; 207 Department of Physics, Tokyo Institute of Technology, Tokyo, Japan; 208 Department of Physics, University of Toronto, Toronto, ON Canada; 209 TRIUMF, Vancouver, BC Canada; 210 Department of Physics and Astronomy, York University, Toronto, ON Canada; 211 Faculty of Pure and Applied Sciences, and Center for Integrated Research in Fundamental Science and Engineering, University of Tsukuba, Tsukuba, Japan; 212 Department of Physics and Astronomy, Tufts University, Medford, MA USA; 213 Department of Physics and Astronomy, University of California Irvine, Irvine, CA USA; 214 INFN Gruppo Collegato di Udine, Sezione di Trieste, Udine, Italy; 215 ICTP, Trieste, Italy; 216 Dipartimento di Chimica Fisica e Ambiente, Università di Udine, Udine, Italy; 217 Department of Physics and Astronomy, University of Uppsala, Uppsala, Sweden; 218 Department of Physics, University of Illinois, Urbana, IL USA; 219 Instituto de Fisica Corpuscular (IFIC) and Departamento de Fisica Atomica, Molecular y Nuclear and Departamento de Ingeniería Electrónica and Instituto de Microelectrónica de Barcelona (IMB-CNM), University of Valencia and CSIC, Valencia, Spain; 220 Department of Physics, University of British Columbia, Vancouver, BC Canada; 221 Department of Physics and Astronomy, University of Victoria, Victoria, BC Canada; 222 Department of Physics, University of Warwick, Coventry, UK; 223 Waseda University, Tokyo, Japan; 224 Department of Particle Physics, The Weizmann Institute of Science, Rehovot, Israel; 225 Department of Physics, University of Wisconsin, Madison, WI USA; 226 Fakultät für Physik und Astronomie, Julius-Maximilians-Universität, Würzburg, Germany; 227 Fakultät für Mathematik und Naturwissenschaften, Fachgruppe Physik, Bergische Universität Wuppertal, Wuppertal, Germany; 228 Department of Physics, Yale University, New Haven, CT USA; 229 Yerevan Physics Institute, Yerevan, Armenia; 230 Centre de Calcul de l’Institut National de Physique Nucléaire et de Physique des Particules (IN2P3), Villeurbanne, France; 231 CERN, 1211 Geneva 23, Switzerland

## Abstract

A study of the decays $B^0_s \rightarrow \mu ^+\mu ^-$ and $B^0 \rightarrow \mu ^+\mu ^-$ has been performed using data corresponding to an integrated luminosity of 25 fb$^{-1}$ of 7 and 8 TeV proton–proton collisions collected with the ATLAS detector during the LHC Run 1. For the $B^0$ dimuon decay, an upper limit on the branching fraction is set at $\mathcal{B}(B^0 \rightarrow \mu ^+\mu ^-) < 4.2 \times 10^{-10}$ at 95 % confidence level. For $B^0_s$, the branching fraction $\mathcal{B}(B^0_s \rightarrow \mu ^+\mu ^-) = \left( 0.9^{+1.1}_{-0.8} \right) \times 10^{-9}$ is measured. The results are consistent with the Standard Model expectation with a *p* value of 4.8 %, corresponding to 2.0 standard deviations.

## Introduction

Flavour-changing neutral-current (FCNC) processes are highly suppressed in the Standard Model (SM), and their study is relevant to indirect searches for physics beyond the SM. The branching fractions of the decays $B^0{}\!_{(s)} \rightarrow \mu ^+\mu ^-$ are of particular interest because of the additional helicity suppression and since they are accurately predicted in the SM: $\mathcal{B}(B^0_s \rightarrow \mu ^+\mu ^-) = (3.65 \pm 0.23) \times 10^{-9}$ and $\mathcal{B}(B^0 \rightarrow \mu ^+\mu ^-) = (1.06 \pm 0.09) \times 10^{-10}$ [1]. Significant deviations from these values can arise in models involving non-SM heavy particles, such as those predicted in the Minimal Supersymmetric Standard Model [2–6], in extensions such as Minimal Flavour Violation [7, 8], Two-Higgs-Doublet Models [6], and others [9, 10]. The CMS and LHCb collaborations have reported the observation of $B^0_s \rightarrow \mu ^+\mu ^-$  [11, 12] and evidence of $B^0 \rightarrow \mu ^+\mu ^-$, with combined values: $\mathcal{B}(B^0_s \rightarrow \mu ^+\mu ^-) = \left( 2.8^{+0.7}_{-0.6}\right) \times 10^{-9}$ and $\mathcal{B}(B^0 \rightarrow \mu ^+\mu ^-) = \left( 3.9^{+1.6}_{-1.4}\right) \times 10^{-10}$ [13].

This paper reports the result of a search for $B^0_s \rightarrow \mu ^+\mu ^-$ and $B^0 \rightarrow \mu ^+\mu ^-$ decays performed using *pp* collision data corresponding to an integrated luminosity of 25 fb$^{-1}$, collected at 7 and 8 TeV in the full LHC Run 1 data-taking period using the ATLAS detector. This analysis supersedes the previous result [14] based on 2011 data and exploits improved analysis techniques in addition to the larger dataset.

## Outline

The $B^0_s \rightarrow \mu ^+\mu ^-$ and $B^0 \rightarrow \mu ^+\mu ^-$ branching fractions are measured relative to the normalisation decay $B^+\rightarrow J/\psi (\rightarrow \mu ^+\mu ^-)K^+$ that is abundant and has a known branching fraction $\mathcal{B}(B^+ \rightarrow J/\psi \,K^+) \times \mathcal{B}(J/\psi \rightarrow \mu ^{+} \mu ^{-})$. In the simplest form, the $B^0 \rightarrow \mu ^+\mu ^-$ ($B^0_s \rightarrow \mu ^+\mu ^-$) branching fraction can be extracted as:

$$\begin{aligned}&\mathcal{B}(B^0_{(s)}\! \rightarrow \!\mu ^+ \mu ^-) \\&\quad = \frac{N_{d(s)}}{\varepsilon _{\mu ^+\mu ^-}}\left[ \mathcal{B}(B^+ \rightarrow J/\psi \,K^+) \times \mathcal{B}(J/\psi \rightarrow \mu ^{+} \mu ^{-})\right] \\&\qquad \times \,\frac{\varepsilon _{J/\psi K^+}}{N_{J/\psi K^+}} \times \frac{f_{u}}{f_{d(s)}}, \end{aligned}$$

where $N_d$ ($N_s$) is the $B^0 \rightarrow \mu ^+\mu ^-$ ($B^0_s \rightarrow \mu ^+\mu ^-$) signal yield, $N_{J/\psi K^+}$ is the $B^+ \rightarrow J/\psi \,K^+$ normalisation yield, $\varepsilon _{\mu ^+\mu ^-}$ and $\varepsilon _{J/\psi K^+}$ are the corresponding values of acceptance times efficiency, and $f_u/f_d$ ($f_u/f_s$) is the ratio of the hadronisation probabilities of a *b*-quark into $B^+$ and $B^0$ ($B_s^0$).

For this study, a modified formula is used to normalise independently samples of events collected in different data-taking periods and with different trigger selections:

1 $$\begin{aligned}&\mathcal{B}(B^0_{(s)}\! \rightarrow \!\mu ^+ \mu ^-) \nonumber \\&\quad = N_{d(s)}\left[ \mathcal{B}(B^+ \rightarrow J/\psi \,K^+) \times \mathcal{B}(J/\psi \rightarrow \mu ^{+} \mu ^{-})\right] \nonumber \\&\qquad \times \, \frac{f_{u}}{f_{d(s)}} \times \frac{1}{\mathcal {D}_{\mathrm {norm}}}, \end{aligned}$$

with

2 $$\begin{aligned} \mathcal {D}_{\mathrm {norm}} = \sum _k N^k_{J/\psi K^+} \alpha _k\left( \frac{\varepsilon _{\mu ^+\mu ^-}}{\varepsilon _{J/\psi \, K^+}} \right) _k. \end{aligned}$$

The denominator $\mathcal {D}_{\mathrm {norm}}$ consists of a sum whose index *k* runs over the data-taking periods and the trigger selections. In the sum, the $\alpha _k$ parameter takes into account the different trigger prescale factors and integrated luminosities in the signal and normalisation channels, and the ratio of the efficiencies corrects for reconstruction differences in each data sample *k*. Signal and reference channel events are selected with similar dimuon triggers.

The notation used throughout the paper refers to both the stated and charge-conjugated process, unless otherwise specified. The analysis is performed without tagging of the flavour $B^0{}\!_{(s)}$ or $\overline{B^0}\!_{(s)}$ at production. The yield measurement in the normalisation channel is obtained by summing $J/\psi K^+$ and $J/\psi K^-$ contributions.

The analysis is performed integrating over the decay time distribution of the event candidates. The relation between the measured branching fraction and the corresponding value at production is established assuming the decay time distribution predicted in the SM, where the decay occurs predominantly through the heavy eigenstate $B_{s/d,\mathrm {H}}$ of the $B^0{}\!_{(s)}$-$\overline{B^0}\!_{(s)}$ system. Models for new physics [15, 16] can predict modification to the decay time distribution of $B^0_s \rightarrow \mu ^+\mu ^-$ and a comparison with the experimental result may require a correction to the ratio of the time-integrated efficiencies.

The ATLAS inner tracking system and muon spectrometer are used to reconstruct and select the event candidates. Details of the detector, trigger, data sets, and preliminary selection criteria are discussed in Sects. 3 and 4. A blind analysis was performed in which data in the dimuon invariant mass region from 5166 to 5526 MeV were removed until the procedures for event selection and the details of signal yield extraction were completely defined. Section 5 introduces the three main categories of background (continuum background due to muons from uncorrelated hadron decays, background from partially reconstructed decays, and peaking background from $B^0{}\!_{(s)}$ two-body hadronic decays, where both particles are misidentified as muon pairs). Section 6 describes the strategy used to reduce the probability of hadron misidentification. The final sample of candidates is selected using a multivariate classifier, designed to enhance the signal relative to the continuum background, as discussed in Sect. 7. Checks on the distributions of the variables used in the multivariate classifier are summarised in Sect. 8. They are based on the comparison of data and simulation for dimuon events, for $B^+ \rightarrow J/\psi \,K^+$ candidates and for events selected as $B^0_s \rightarrow J/\psi \, \phi \rightarrow \mu ^+\mu ^- K^+K^-$, which provide an additional validation of the procedures used in the analysis. Section 9 details the fit procedure to extract the yield of $B^+ \rightarrow J/\psi \,K^+$ events. As an ancillary measurement to the $B^+ \rightarrow J/\psi \,K^+$ yield determination, a measurement of the ratio $\mathcal {B}(B^+ \rightarrow J/\psi \,\pi ^+ )/\mathcal {B}(B^+ \rightarrow J/\psi \,K^+)$ is performed, as presented in Sect. 9.1. The ratio of efficiencies in the signal and the normalisation channels is presented in Sect. 10. Section 11 describes the extraction of the signal yield, obtained with an unbinned maximum-likelihood fit performed on the dimuon invariant mass distribution, with the events classified according to three intervals in the classifier used for the final selection. The results on the branching fractions $\mathcal B$($B^0_s \rightarrow \mu ^+\mu ^-$) and $\mathcal B$($B^0 \rightarrow \mu ^+\mu ^-$) are reported in Sect. 12.

## ATLAS detector, data and simulation samples

The ATLAS detector^1^ consists of three main components: an inner detector (ID) tracking system immersed in a 2 T axial magnetic field, surrounded by electromagnetic and hadronic calorimeters and by the muon spectrometer (MS). A full description can be found in Ref. [17].

This analysis is based on the Run 1 data sample recorded in 2011 and 2012 by the ATLAS detector from *pp* collisions at the LHC at $\sqrt{s}=7$ and 8 TeV, respectively. Data used in the analysis were recorded during stable LHC beam periods. Data quality requirements were imposed, notably on the performance of the MS and ID systems. The total integrated luminosity of good quality data used in this analysis is 4.9 fb$^{-1}$ for the 2011 sample and 20 fb$^{-1}$ for 2012. The average number of reconstructed primary vertices (PV) per event, related to multiple proton–proton interactions, is 6.2 and 11.4 in the two years respectively.

Samples of simulated Monte-Carlo (MC) events are used for training and validation of the multivariate analyses, for the determination of the efficiency ratios, and for guiding the signal extraction fits. Exclusive MC samples were produced for the signal channels $B^0_s \rightarrow \mu ^+\mu ^-$ and $B^0 \rightarrow \mu ^+\mu ^-$, the normalisation channel $B^+ \rightarrow J/\psi \,K^+$ ($J/\psi \rightarrow \mu ^+\mu ^-$), the $B^+ \rightarrow J/\psi \, \pi ^+$ channel, and the control channel $B^0_s \rightarrow J/\psi \, \phi$ ($\phi \rightarrow K^+K^-$). In addition, background studies employ MC samples of inclusive semileptonic decays $B\rightarrow \mu X$, samples of $B_s^0 \rightarrow K^- \mu ^+ \nu$, $B^0 \rightarrow \pi ^-\mu ^+ \nu$, $\Lambda _b \rightarrow p \mu ^- \overline{\nu }$, $B^0{}\!_{(s)} \rightarrow hh^\prime$ decays with $h^{(\prime )}$ being a charged pion or kaon, and inclusive decays $B \rightarrow J/\psi X$.

Most of dimuon candidates in the data sample originate from the uncorrelated decays of hadrons produced in the hadronisation of a *b* and a $\bar{b}$ quarks. To describe this background, defined as continuum, a large MC sample was generated by selecting specific topologies that dominate it. The strategy is to consider both the primary decays from *b* quarks and the secondary decays from *c* quarks. Independent samples of events with forced semileptonic decays or decays including muons pairs from $J/\psi$ were generated in all combinations. The total number of events in each sample is chosen to reproduce the composition of oppositely charged muon pairs representative of our data.

The MC samples were generated with Pythia 6 [18] for studies related to data collected in 2011, and with Pythia 8 [19] and EvtGen [20] for the 2012 sample and the development of multivariate classifiers. The ATLAS detector and its response are simulated using Geant4 [21, 22]. Additional *pp* interactions in the same and nearby bunch crossings (pile-up) are included in the simulation. All simulated samples are reweighted to have the same distribution of the number of PVs per bunch crossing found in data.

Using the iterative reweighting method described in Ref. [14], the simulated samples of the exclusive decays considered are adjusted with two-dimensional data-driven weights (DDW) to correct for the differences between simulation and data observed in the $p_{\text {T}} ^B$ and and $|\eta ^B|$ distributions. DDW obtained from $B^+ \rightarrow J/\psi \,K^+$ decays are used to correct the simulation samples in the signal and normalisation channels. DDW obtained from the $B^0_s \rightarrow J/\psi \, \phi$ control channel are found to agree with those from $B^+ \rightarrow J/\psi \,K^+$ showing the consistency of the corrections.

Similarly to the exclusive decays, the large continuum background MC sample is reweighted via DDW obtained from its comparison with the data in the sidebands of the signal region.

## Data selection

For data collected during the LHC Run 1, the ATLAS detector used a three-level trigger system, consisting of a hardware-based Level-1 trigger, software-based Level-2 and Event Filter triggers.

A dimuon trigger [23, 24] is used to select events. The 2011 data sample contains events seeded by a Level-1 dimuon trigger that required a transverse momentum $p_{\text {T}} > 4$ GeV for both muon candidates. Due to the increased pile-up in 2012 data, this dimuon trigger was prescaled at the beginning of every fill. The effect of prescaling is mitigated by including in the analysis events selected by two additional Level-1 triggers scarcely affected by prescaling, where tighter selections were applied: $p_{\text {T}} > 6$ GeV or $|\eta | < 1.05$ for one of the muons. A full track reconstruction of the muon candidates was performed at the software trigger levels, where an additional loose selection was applied to the dimuon invariant mass $m_{\mu \mu }$ and the events were assigned to the $J/\psi$ stream ($2.5< m_{\mu \mu } < 4.3$ GeV) or to the *B* stream ($4.0< m_{\mu \mu } < 8.5$ GeV).

Events from the 2012 dataset are divided into three mutually exclusive trigger categories:

- $T_1$  “Higher threshold” trigger with $p_{\text {T}}$ $>6$ GeV for one muon and $>4$ GeV for the other one;
- $T_2$  “Barrel” trigger with $p_{\text {T}}$ $>4$ GeV for both muon candidates and at least one of them with $|\eta | < 1.05$ (and $T_1$ requirement not satisfied);
- $T_3$  Basic dimuon trigger with $p_{\text {T}}$ $>4$ GeV for both muon candidates (and $T_1$, $T_2$ requirements not satisfied).

Events belonging to a given category are all associated with the same pattern of Level-1 prescaling. The event sample in the $T_2$ ($T_3$) category has an equivalent integrated luminosity equal to $97.7\,\%$ ($81.3\,\%$) of the luminosity of the $T_1$ category. The impact of the trigger Level-1 prescale on the total sample of collected events is minor, since the majority of the events belong to the $T_1$ category.

The events in the reference channels $B^+ \rightarrow J/\psi \,K^+$ and $B_s^0 \rightarrow J/\psi \phi$ collected in 2012 belong to a prescaled sample of events, which was processed together with the signal events. The effective prescaling factor is equal to 7.3, and does not affect the sensitivity of this analysis, given the large number of available events in the normalisation channel. This factor is included in the $\alpha _k$ parameters in Eq. (1).

A fourth category is defined for events from the 2011 dataset. They were collected with a trigger requirement $p_{\text {T}}$ $>4$ GeV for both muon candidates, and prescaling was not applied to this sample.

After off-line reconstruction, a preliminary selection is performed on candidates for $B^0{}\!_{(s)} \rightarrow \mu ^+\mu ^-$, $B^+ \rightarrow J/\psi \,K^+ \rightarrow \mu ^{+} \mu ^{-} K^+$ and $B_s^0$ $\rightarrow J/\psi \phi \rightarrow \mu ^{+} \mu ^{-} K^+ K^-$. In the ID system, muons are required to have at least one hit in the pixel detector, five hits in the semiconductor tracker (two hits per each double-sided layer), and six hits in the transition-radiation tracker, if $0.1< |\eta |< 1.9$. They are also required to be reconstructed in the MS, and to have $|\eta | < 2.5$ and $p_{\text {T}} >4$ GeV. Kaon candidates have to satisfy similar requirements in the ID, except that at least nine instead of six hits are required in the transition-radiation tracker and a looser requirement of $p_{\text {T}} >1$ GeV is imposed.

*B* meson properties are computed based on a decay vertex fitted to two, three or four tracks, depending on the decay process to be reconstructed. The $\chi ^2$ per degree of freedom in the vertex fit is required to be less than six for the *B* vertex, and less than ten for the $J/\psi \rightarrow \mu \mu$ vertex. The conditions $2915<m(\mu \mu )<3275$ MeV and $1005< m(KK) < 1035$ MeV are required on ID track combinations for the $J/\psi \rightarrow \mu \mu$ and the $\phi \rightarrow K K$ vertices, respectively. In the $B^+ \rightarrow J/\psi \,K^+$ and $B^0_s \rightarrow J/\psi \, \phi$ fits the reconstructed $J/\psi$ mass is constrained to the world average value [25].

Reconstructed *B* candidates are required to satisfy $p_{\text {T}} ^B>8.0$ GeV and $|\eta ^B|<2.5$. The dimuon invariant mass for $B^0{}\!_{(s)} \rightarrow \mu ^+\mu ^-$ candidates is calculated using the combined ID and MS information, in order to improve the mass resolution in the end-caps with respect to using ID information only [26].

The invariant mass range considered for the $B^0{}\!_{(s)} \rightarrow \mu ^+\mu ^-$ decay is 4766–5966 MeV in which the 5166–5526 MeV range is defined as the signal region while the low-mass and high-mass regions (4766–5166 and 5526–5966 MeV) are the signal mass sidebands. For the reference channels, the mass range considered is 4930–5630 (5050–5650) MeV for $B^+ \rightarrow J/\psi \,K^+$ ($B^0_s \rightarrow J/\psi \, \phi$) in which the 5180–5380 (5297–5437) MeV range is the peak region and the two low and high mass ranges are the mass sidebands used for background subtraction.

The coordinates of the PVs are obtained from charged tracks not used in the decay vertices, and are transversely constrained to the luminous region of the colliding beams. The matching of a *B* candidate to a PV is made by propagating the candidate to the point of closest approach to the collision axis, and choosing the PV with the smallest separation along *z*. Simulation shows that this method achieves a correct matching probability of better than $99\,\%$.

To reduce of the large background in the $B^0{}\!_{(s)} \rightarrow \mu ^+\mu ^-$ channel before the final selection based on multivariate classifiers, a loose collinearity requirement is applied between the momentum of the *B* candidate ($\overrightarrow{p}^B$) and the spatial separation between the PV and the decay vertex ($\overrightarrow{\Delta x}$). The absolute value of the difference in azimuthal angle $\alpha _{2\mathrm {D}}$ is required to be smaller than 1.0 rad. Using the difference in rapidity $\Delta \eta$, the combination $\Delta R = \sqrt{\alpha _{2\mathrm {D}}{}^{2} + \Delta \eta ^{2}}$ is required to be smaller than 1.5. These requirements reduce the background by a factor of 0.4, with a signal efficiency of $95\,\%$.

After the preliminary selection, approximately $2.6\times 10^6$ ($2.3 \times 10^6$) candidates are found in the $B^0{}\!_{(s)} \rightarrow \mu ^+\mu ^-$ ($B^+ \rightarrow J/\psi \,K^+$) signal regions.

## Background composition

The background to the $B^0{}\!_{(s)} \rightarrow \mu ^+\mu ^-$ signal originates from three main sources:

*Continuum*: Background, the dominant combinatorial component, made from muons coming from uncorrelated hadron decays and characterised by a small dependence on the dimuon invariant mass;
*Partially reconstructed*: $B\rightarrow \mu \mu X$ decays, characterised by non-reconstructed final-state particles (*X*) and thus accumulating in the low dimuon invariant mass sideband;
*Peaking*: Background, due to $B^0{}\!_{(s)}\rightarrow h\,h^\prime$ decays, with both hadrons misidentified as muons.

The continuum background consists mainly of muons independently produced in the fragmentation and decay trees of a *b* and a $\overline{b}$ quark (*opposite-side* muons). It is studied in the signal mass sidebands, and it is found to be correctly described by the inclusive MC sample of semileptonic decays of *b* and *c* hadrons.

Section 8 contains data–MC comparisons for the continuum background. As discussed in Sect. 7, a multivariate classifier trained on MC samples is used to reduce this component.

The partially reconstructed decays consist of several topologies: (a) *same-side* (SS) combinatorial background from decay cascades ($b \rightarrow c \mu \nu \rightarrow s(d) \mu \mu \nu \nu$); (b) *same-vertex* (SV) background from *B* decays containing a muon pair (e.g. $B^0 \rightarrow K^{*0}\mu \mu$, $B \rightarrow J/\psi X \rightarrow \mu \mu \mu X^\prime$); (c) $B_c$ decays (e.g. $B_c \rightarrow J/\psi \mu \nu \rightarrow \mu \mu \mu \nu$); (d) semileptonic *b*-hadron decays where a final-state hadron is misidentified as a muon.

Inclusive MC samples of SS events, SV events, and $B_c \rightarrow J/\psi \mu \nu$ decays were generated. All subsamples have a dimuon invariant mass distribution accumulating below the mass range considered in this analysis. The high-mass tail extends to the signal region and becomes a significant fraction of the background only after applying a selection against the continuum background.

**Fig. 1:**
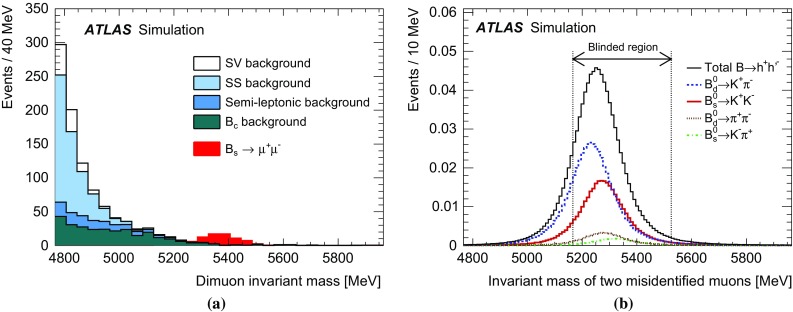
**a** Dimuon invariant mass distribution for the partially reconstructed background, from simulation, before the final selection against continuum is applied but after all other requirements. The different components are shown as stacked histograms, normalised according to world-averaged measured branching fractions. The SM expectation for the $B^0_s \rightarrow \mu ^+\mu ^-$ signal is also shown for comparison (non-stacked). Continuum background is not included here. **b** Invariant mass distribution of the peaking background components $B^0{}\!_{(s)} \rightarrow hh^\prime$, after the complete signal selection is applied. In both plots the distributions are normalised to the expected yield for the integrated luminosity of 25 fb$^{-1}$.

The semileptonic decays with final-state hadrons misidentified as muons consist mainly of three-body charmless decays $B^0 \rightarrow \pi \mu \nu$, $B^0_s \rightarrow K\mu \nu$ and $\Lambda _b \rightarrow p\mu \nu$ in which the tail of the invariant mass distribution extends to the signal region. Due to branching fractions of the order of $10^{-6}$, this background is not large, and is further reduced by the dedicated muon identification requirements, discussed in Sect. 6. The MC invariant mass distributions of these partially reconstructed decay topologies are shown in Fig. 1a after applying the preliminary selection criteria described in Sect. 4.

Finally, the peaking background is due to $B^0{}\!_{(s)}$ decays containing two hadrons misidentified as muons, which populate the signal region as shown in Fig. 1b.

## Hadron misidentification

In the preliminary selection, muon candidates are formed from the combination of tracks reconstructed independently in the ID and MS [27]. The performance of the muon reconstruction in ATLAS is presented in Ref. [26]. Additional studies were performed for this analysis to minimise and evaluate the amount of background related to hadrons erroneously identified as muons.

Detailed simulation studies were performed for the channels $B^0{}\!_{(s)} \rightarrow hh^\prime$ and $\Lambda _b \rightarrow ph$, with $h^{(\prime )} = \pi ^\pm , K^\pm$. A full Geant4-based simulation [21] in all systems of the ATLAS detector is used for this purpose. The vast majority of background events from particle misidentification are due to decays in flight of kaons and pions, in which the muon receives most of the energy of the meson. Hence, despite the notation of *fake* muons, this background is generally related to true muons measured in the MS, but not produced promptly in the decay of a *B* meson. The contribution from hadronic punch-through into the MS is expected from simulation to amount only to 3 % (8 %) of the total number of fake candidates from kaons (pions).

The simulation shows that after the preliminary selection the probability for a kaon (pion) to be misidentified as a muon is 0.4 % (0.2 %). This fraction is found to be largely independent of the transverse momentum and rapidity of the track, as well as other variables related to the underlying event or pile-up. The misidentification rate for protons is found to be negligible (${<}0.01~\%$).

The muon candidate is further required to match the trigger requirements, resulting in a reduction in the number of retained tracks by a factor 0.58, and to pass an additional multivariate selection, implemented as a boosted decision tree (BDT) [28]. This selection, referred to as *fake-BDT*, is based on variables described in Table 1 and it is built and trained on the MC samples. The BDT training is done using a multivariate analysis tool (TMVA) [28]. The fake-BDT selection is tuned for a 95 % efficiency for muons in the signal sample, and achieves an average reduction of the hadron misidentification by a factor 0.37, determined with independent MC samples. The resulting final value of the misidentification probability is equal to 0.09 % for kaons and 0.04 % for pions.

**Table 1:** Description of the eight variables used in the discrimination between signal muons and those from hadron decays in flight and punch-throughs

1.	Absolute value of the track rapidity measured in the ID
2.	Ratio *q* / *p* (charge over momentum) measured in the MS
3.	Scattering curvature significance: maximum value of the significance of the track curvature variation across each layer of the ID
4.	$\chi ^2$ of the track reconstruction in the MS
5.	Number of hits used to reconstruct the track in the MS
6.	Ratio of the values of *q* / *p* measured in the ID and in the MS, corrected for the average energy loss in the calorimeter
7.	$\chi ^2$ of the match between the tracks reconstructed in the ID and MS
8.	Energy deposited in the calorimeters along the muon trajectory obtained by combining ID and MS tracks

The background due to $B^0{}\!_{(s)} \rightarrow hh^\prime$, with double misidentification $h h^\prime \rightarrow \mu \mu$, has a distribution in the reconstructed invariant mass peaking at 5250 MeV, close to the $B_s^0$ mass and is effectively indistinguishable from the $B^0$ signal (see Fig. 1b). Beyond the muon and fake-BDT selection, these events have the same acceptance and selection efficiency as the $B^0{}\!_{(s)} \rightarrow \mu ^+\mu ^-$ signal. Therefore, the expected number of peaking-background events can be estimated from the number of observed $B^+ \rightarrow J/\psi \,K^+$ events, in a way analogous to what is done for the signal, using Eq. (1). World average [25] values for the branching fractions of $B^0$ and $B_s^0$ into $K\pi$, *KK* and $\pi \pi$ are used, together with the hadron misidentification probabilities obtained from simulation. The resulting total expected number of peaking-background events, after the final selection (including a multivariate cut against $\mu ^+\mu ^-$ continuum background, the *continuum*-BDT discussed in Sect. 7), is equal to 0.7, with a 10 % uncertainty from the normalisation procedure.

The simulation of hadron misidentification was validated and calibrated with studies performed on data. The fractions of fake muons after the preliminary selection were evaluated on samples of $\phi \rightarrow K^+K^-$ and $B^+ \rightarrow J/\psi \,K^+$ events, and found to be consistent with the simulation within a factor $1.2\pm 0.2$. This factor and its square $1.4 \pm 0.5$ are used as scale correction and systematic uncertainty in the single and double misidentification probability, respectively. Hence, the expected number of peaking background events is equal to $1.0\pm 0.4$.

A further test of the peaking background was performed on the final sample of $B^0{}\!_{(s)} \rightarrow \mu ^+\mu ^-$ candidates. Inverting the selection applied with the fake-BDT, the number of events containing real muons is largely reduced, while the number of peaking-background events is approximately three times larger than in the sample obtained with the nominal selection. A fit to the background-enhanced sample gives a peaking background yield of $0.5 \pm 3.0$ events, in good agreement with the expectation.

The efficiency of the fake-BDT selection when applied to muons from $B^0{}\!_{(s)} \rightarrow \mu ^+\mu ^-$ decays was tested on the sample of $B^+ \rightarrow J/\psi \,K^+$ candidates selected in data. The value from simulation was found to be accurate to better than $1~\%$.

Besides the peaking background, the selection with the fake-BDT also reduces the semileptonic contributions with a single misidentified hadron. The expected number of events from $B^0 \rightarrow \pi \mu \nu$ and $B_s^0 \rightarrow K\mu \nu$ in the final sample is $107 \pm 27$. The $\Lambda _b \rightarrow p\mu \nu$ contribution is negligible due to the smaller production cross section and the fake rejection for protons at the level of $10^{-5}$.

## Continuum background reduction

A multivariate analysis, implemented as a BDT, is employed to enhance the signal relative to the continuum background. This classifier, referred to as the *continuum-BDT*, is based on the 15 variables described in Table 2. The discriminating variables can be classified into three groups: (a) *B* meson variables, related to the reconstruction of the decay vertex and to the collinearity between $\overrightarrow{p}^B$ and the separation between production and decay vertices $\overrightarrow{\Delta{x}}$; (b) variables describing the muons forming the *B* meson candidate; and (c) variables related to the rest of the event. The selection of the variables aims to optimise the discrimination power of the classifier, while minimising the dependence on the invariant mass of the muon pair.

Most of the discriminating variables are part of the set used in the previous analysis based on data collected in 2011 [14], while others were modified or added, exploiting the statistical power of the large samples of MC events used for training and validating the classifier. To minimise the dependence of the classifier on the effects of the pile-up, requirements of compatibility with the same vertex matched to the dimuon candidate are placed on the additional tracks considered for the variables $I_{0.7}$, DOCA$_{\mathrm {xtrk}}$ and $N^{\mathrm {close}}_{\mathrm {xtrk}}$.

The correlation between the discriminating variables was studied in the MC samples for signal and continuum background discussed in Sect. 3, and on data from the sidebands of the $\mu ^+\mu ^-$ invariant mass distribution. Different degrees of correlation are present, with significant linear correlation among the variables $\chi ^{2}_{{\mathrm {PV,DV}}\,xy}$, $L_{xy}$, $|d_{0}|^{\mathrm {max}}$-sig., $|d_{0}|^{\mathrm {min}}$-sig. and $\chi ^2_{\mu ,\mathrm {xPV}}$. Conversely, the variables IP$_B^{3\mathrm {D}}$, DOCA$_{\mu \mu }$ and $I_{0.7}$ have negligible correlation with any of the others used in the classifier.

**Table 2:** Description of the 15 variables used in the discrimination between signal and continuum background. When the BDT classifier is applied to $B^+ \rightarrow J/\psi \,K^+$ and $B^0_s \rightarrow J/\psi \, \phi$ candidates, the variables related to the decay products of the *B* mesons refer only to the muons from the decay of the $J/\psi$

Variable	Description
$p_{\text {T}} ^{B}$	Magnitude of the *B* candidate transverse momentum $\overrightarrow{p_{\text {T}}}^B$
$\chi ^{2}_{{\mathrm {PV,DV}}\;xy}$	Significance of the separation $\overrightarrow{\Delta x}$ between production (*i.e.* associated PV) and decay (DV) vertices in the transverse projection: $\overrightarrow{\Delta x}_\text {T}\!\cdot \! \Sigma _{\overrightarrow{\Delta x}_\text {T}}^{\phantom {x}-1}\!\cdot \!\overrightarrow{\Delta x}_\text {T}$, where $\Sigma _{\overrightarrow{\Delta x}_\text {T}}$ is the covariance matrix
$\Delta {R}$	Three-dimensional opening between $\overrightarrow{p}^{B}$ and $\overrightarrow{\Delta x}$: $\sqrt{\alpha _{2\mathrm {D}}{}^{2} + \Delta \eta ^{2}}$
$|\alpha _{2\mathrm {D}}|$	Absolute value of the angle between $\overrightarrow{p_{\text {T}}}^{B}$ and $\overrightarrow{\Delta x}_\text {T}$ (transverse projection)
$L_{xy}$	Projection of $\overrightarrow{\Delta x}_\text {T}$ along the direction of $\overrightarrow{p}_\text {T}^{B}$: $(\overrightarrow{\Delta x}_\text {T}\!\cdot \!\overrightarrow{p_{\text {T}}}^{B})/|\overrightarrow{p_{\text {T}}}^{B}|$
IP$_B^{3\mathrm {D}}$	Three-dimensional impact parameter of the *B* candidate to the associated PV
DOCA$_{\mu \mu }$	Distance of closest approach (DOCA) of the two tracks forming the *B* candidate (three-dimensional)
$\Delta \phi _{\mu \mu }$	Difference in azimuthal angle between the momenta of the two tracks forming the *B* candidate
$|d_{0}|^{\mathrm {max}}$-sig.	Significance of the larger absolute value of the impact parameters to the PV of the tracks forming the *B* candidate, in the transverse plane
$|d_{0}|^{\mathrm {min}}$-sig.	Significance of the smaller absolute value of the impact parameters to the PV of the tracks forming the *B* candidate, in the transverse plane
$P_{\text {L}}^{\mathrm {min}}$	Value of the smaller projection of the momenta of the muon candidates along $\overrightarrow{p_{\text {T}}}^B$
$I_{0.7}$	Isolation variable defined as ratio of $|\overrightarrow{p_{\text {T}}}^{B}|$ to the sum of $|\overrightarrow{p_{\text {T}}}^{B}|$ and of the transverse momenta of all additional tracks contained within a cone of size $\Delta {R} < 0.7$ around the *B* direction. Only tracks with $p_{\text {T}} > 0.5$ GeV and matched to the same PV as the *B* candidate are included in the sum
DOCA$_{\mathrm {xtrk}}$	DOCA of the closest additional track to the decay vertex of the *B* candidate. Tracks matched to a PV different from the *B* candidate are excluded
$N^{\mathrm {close}}_{\mathrm {xtrk}}$	Number of additional tracks compatible with the decay vertex (DV) of the *B* candidate with $\ln (\chi ^2_{\mathrm {xtrk,DV}})\! < \! 1$. The tracks matched to a PV different from the *B* candidate are excluded
$\chi ^2_{\mu ,\mathrm {xPV}}$	Minimum $\chi ^2$ for the compatibility of a muon in the *B* candidate with a PV different from the one associated with the *B* candidate

The MC sample for signal and the large MC sample of semileptonic decays of hadrons containing *b* or *c* quarks are used for training and testing the classifier. As discussed in Sect. 3, signal and background samples are reweighted according to the distributions of $p_{\text {T}}$ and $|\eta |$ of the dimuon and of the number of reconstructed PVs observed in data. To reproduce accurately the 2012 data distributions, MC events belonging to different trigger streams are reweighted according to the relative equivalent luminosity and to two different versions of the Level-2 muon reconstruction algorithm used during the data taking. The BDT training is done using TMVA [28].

**Fig. 2:**
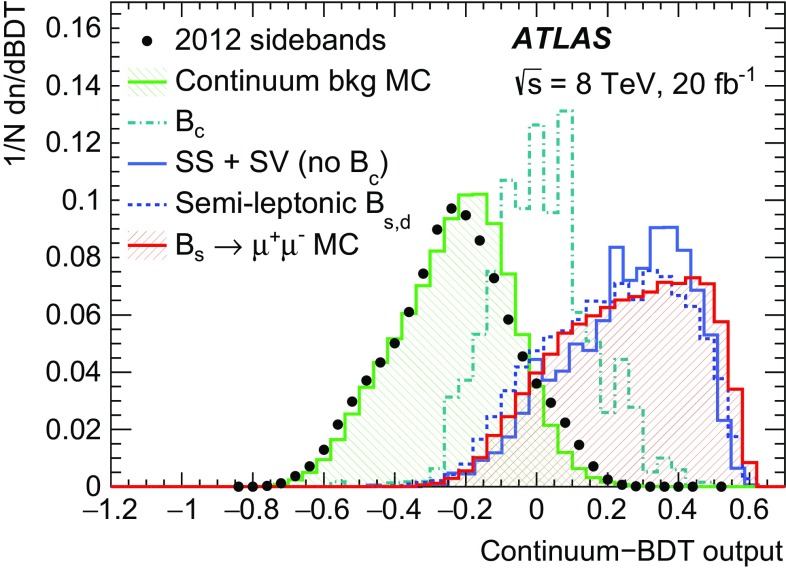
Continuum-BDT distribution for the signal and background events: signal $B^0{}\!_{(s)}$, partially reconstructed *B* events (SS+SV), $B_c$ decays and continuum. The solid histograms are obtained from simulation, while the points represent data collected in the sidebands. All distributions are normalised to unity. The distributions are shown after the preliminary selection, and before applying any reweighting to the variables used in the classifier

Figure 2 shows the distribution of the BDT output variable for signal and background, separately for continuum background and partially reconstructed events. Also shown is the BDT distribution for dimuon candidates from data, from the sidebands of the invariant mass distribution. In both the signal and background MC samples, the absolute value of the linear correlation coefficient between the BDT output and the dimuon invariant mass is smaller than 1 %. The final selection requires a continuum-BDT output value larger than 0.24, corresponding to a signal relative efficiency of $54~\%$ (see Sect. 11), and to a reduction of the continuum background by a factor of about $10^{-3}$.

## Data–simulation comparisons

**Fig. 3:**
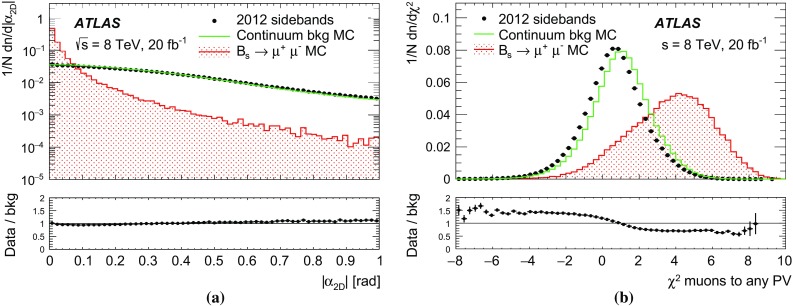
Data and continuum MC distributions of $|\alpha _{2\mathrm {D}}|$ (**a**) and $\chi ^2{}_{\mu ,\mathrm {xPV}}$ (**b**) variables (see Table 2). The *dots* correspond to the 2012 sideband data, while the *continuous-line* histogram corresponds to the continuum MC distribution, normalised to the number of data events. The *filled-area* histogram shows the signal MC distribution for comparison. Discrepancies between MC events and sideband data like the one observed for $\chi ^2_{\mu ,\mathrm {xPV}}$ do not compromise significantly the optimisation of the continuum-BDT classifier

The distributions of the discriminating variables are used to compare the MC sample of semileptonic decays with data in the dimuon sidebands. Figure 3 shows the distributions for two discriminating variables. Agreement with the sideband data is fair and the discrepancies observed do not compromise the use of this MC background sample for the purpose of training the continuum-BDT. The continuum MC simulation is not used for computation of efficiencies or normalisation purposes.

The distributions of the discriminating variables are also used for the comparison of $B^+ \rightarrow J/\psi \,K^+$ and $B^0_s \rightarrow J/\psi \, \phi$ events between simulation and data. To perform such comparison, for each variable the contribution of the background is subtracted from the signal. For this purpose, a maximum-likelihood fit is performed to the invariant mass distribution, separately in the four trigger and data categories. For $B^+$, the signal is described by two overlying Gaussian distributions, an error function for the partially reconstructed decays and an exponential function for the continuum background. The fit model is simpler than the one used for the extraction of the $B^+$ signal used for normalisation after the final selection, described in Sect. 9, but it is sufficient for the purpose discussed here. For $B^0_s \rightarrow J/\psi \, \phi$, a Gaussian distribution is used for the signal and a third-order Chebychev polynomial for the background. For each discriminating variable, the background distribution observed in the sidebands is interpolated to the signal region, normalised according to the result of the likelihood fit, and subtracted from the distribution observed in the signal region.

**Fig. 4:**
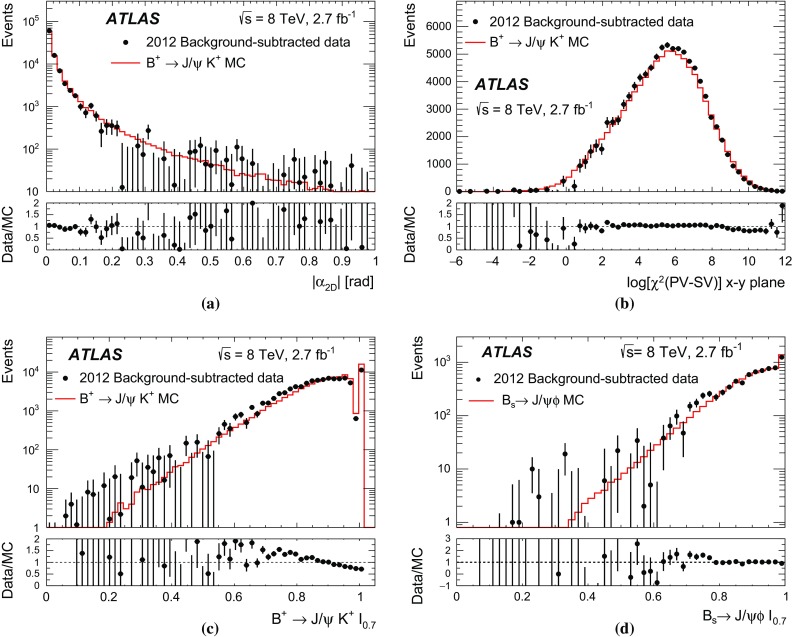
Data and MC distributions in $B^+ \rightarrow J/\psi \,K^+$ events for the discriminating variables: $|\alpha _{2\mathrm {D}}|$ (**a**), $\chi ^{2}{}_{{\mathrm PV,DV}\,xy}$ (**b**) and $I_{0.7}$ (**c**). The variable $I_{0.7}$ is also shown for $B^0_s \rightarrow J/\psi \, \phi$ events (**d**). The *black dots* correspond to the sideband-subtracted data, while the *red* histogram corresponds to the MC distribution, normalised to the number of data events. Differences in shape between MC events and data are accounted for as systematic effects. The discrepancy shown for $I_{0.7}$ in the $B^+ \rightarrow J/\psi \,K^+$ channel is the most significant among all variables and both reference channels

Figure 4 shows examples of the distributions of the discriminating variables obtained from data and simulation. In general, the overall shapes of distributions are in good agreement between data and MC events. Observed differences are accounted for as systematic effects with the procedure described in Sect. 10. The discrepancy shown for the isolation variable $I_{0.7}$ in the $B^+ \rightarrow J/\psi \,K^+$ channel is the most significant one among all variables and both reference channels.

## Yield extraction for the normalisation channel $B^+ \rightarrow J/\psi K^+$

The $B^+$ yield for the normalisation channel is extracted with an unbinned extended maximum-likelihood fit to the $J/\psi K^+$ invariant mass distribution. The functional forms used to model both the signal and the backgrounds are obtained from studies of MC samples. All the yields are extracted from the fit to data, while the shape parameters are determined from a simultaneous fit to data and MC samples. Free parameters are introduced for the mass scale and mass resolution to accommodate data–MC differences.

**Fig. 5:**
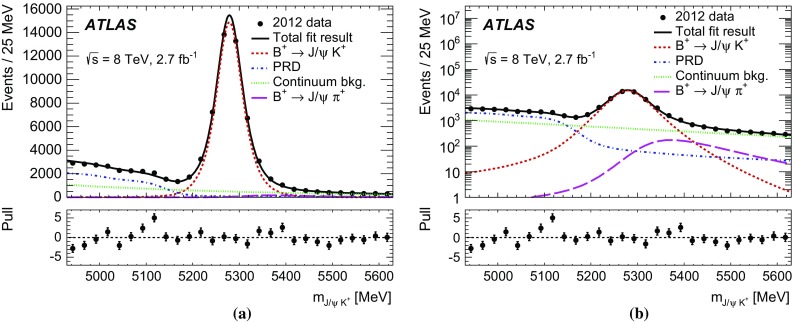
$J/\psi K^+$ invariant mass distribution for all $B^+$ candidates in the $T_1$ trigger category in 2012 data in linear (**a**) and logarithmic (**b**) scale. The result of the fit is overlaid. The various components of the spectrum are described in the text. The *insets* at the *bottom* of the plots show the bin-by-bin pulls for the fits, where the pull is defined as the difference between the data point and the value obtained from the fit function, divided by the error from the fit

The fit includes four components: $B^+ \rightarrow J/\psi \,K^+$ events, Cabibbo-suppressed $B^+ \rightarrow J/\psi \, \pi ^+$ events on the right tail of the main peak, partially reconstructed *B* decays (PRD) where one or more of the final-state particles are missing, and the continuum background composed mostly of $b\bar{b}\rightarrow J/\psi X$ events. The shape of the $B^+ \rightarrow J/\psi \,K^+$ distribution is parameterised using a Johnson $S_U$ function [29, 30] and a Gaussian function for the $T_1$, $T_2$ and 2011 categories, while a single Johnson $S_U$ function is used for the $T_3$ category. The final $B^+ \rightarrow J/\psi \,K^+$ yield includes the contribution from radiative decays. The $B^+ \rightarrow J/\psi \, \pi ^+$ events are modelled by the sum of a Johnson $S_U$ and a Gaussian function, where all parameters are determined from the simulated data. The PRD are described with combinations of Fermi–Dirac and exponential functions, slightly different between the different categories in the low-mass region. Their shape parameters are determined from simulation. Finally, the continuum background is modelled with an exponential function with the shape parameter extracted from the fit. As an example, the fit for the $T_1$ category is shown in Fig. 5. The results of the fits in all data categories are shown in Table 3.

Some of the systematic effects are included automatically in the fit: the effect of limited MC sample size, for example, is included in the uncertainties through a simultaneous fit to data and MC samples. Scaling factors determined in the fit to data account for the differences in mass scale and resolution between data and simulation. Additional systematic uncertainties are evaluated by varying the default fit model described above: they take into account the kinematic differences between data and the MC samples used in the fit, differences in efficiency between $B^+$ and $B^-$ decays, uncertainties in the relative fractions and shapes of PRD, and in the shape of the continuum background. In each case, the difference with respect to the default fit is recorded, symmetrised and used as an estimate of the systematic uncertainty. The main contributions to the systematic uncertainty come from the shape of the continuum background, the relative fractions of PRD and the signal charge asymmetry. The total statistical and systematic uncertainty in the $B^+$ normalisation yield amounts to $0.8~\%$.

### $B^+ \rightarrow J/\psi \, \pi ^+$ / $B^+ \rightarrow J/\psi \,K^+$ branching fraction ratio measurement

For further validation of the fit to the $B^+ \rightarrow J/\psi \,K^+$ yield, the fit described in Sect. 9 is used to extract the yields for $B^+ \rightarrow J/\psi \,K^+$ and $B^+ \rightarrow J/\psi \, \pi ^+$ decays and obtain the ratio $\rho _{\pi /K}$ of the corresponding branching fractions. The measurement is performed separately in the four categories, and combined into an uncertainty-weighted mean ${\rho _{\pi /K}}$. Table 3 shows the fitted yields.

Most systematic effects cancel in the measurement of this ratio. Residual systematic uncertainties in the ratio of the branching fractions come from the uncertainties in the $K^{-}/K^{+}$, $\pi ^{-}/\pi ^{+}$ and $K^{+}/\pi ^{+}$ relative efficiencies. For each systematic effect the ratio is re-evaluated, therefore accounting for correlated effects. The largest systematic uncertainty in the measured ratio comes from the continuum background model parameterisation ($23~\%$), followed by the effect of the uncertainties in the PRD fraction estimates ($15~\%$). All other systematic sources have uncertainties at the level of $10~\%$ or less. The final result for the ratio of branching fractions is:

$$\begin{aligned} {\rho _{\pi /K}} = \frac{\mathcal{B}(B^{+} \rightarrow J/\psi \pi ^{+} )}{\mathcal{B}(B^{+} \rightarrow J/\psi K^{+}) } = 0.035 \pm 0.003 \pm 0.012 \,, \end{aligned}$$

where the first uncertainty is statistical and the second is systematic. The result is in agreement with the most accurate available results from LHCb ($0.0383 \pm 0.0011 \pm 0.0007$ [31]) and BABAR ($0.0537 \pm 0.0045 \pm 0.0011$ [32]).

## Evaluation of the $B^+ \rightarrow J/\psi \,K^+$ to $B^0{}\!_{(s)}\rightarrow \mu ^{+} \mu ^{-}$ efficiency ratio

The ratio of efficiencies for $B^+ \rightarrow J/\psi \,K^+$ and $B^0{}\!_{(s)} \rightarrow \mu ^+\mu ^-$ enters the $\mathcal {D}_{\mathrm {norm}}$ term defined in Eq. (2). Both channels are measured in the fiducial volume of the *B* meson defined as $p_{\text {T}} ^B>8.0$ GeV and $\left| \eta _B\right| <2.5$.

**Table 3:** Results of the fits to the events reconstructed as $B^+ \rightarrow J/\psi \,K^+$ in each trigger and data category. Uncertainties are statistical and systematic, respectively

Category	$N_{J/\psi K^+}$	$N_{J/\psi \pi ^+}$
$T_1$	$46\,860 \pm 290 \pm \,280$	$1\,420 \pm 230 \pm \,440$
$T_2$	$5\,200 \pm 84 \pm \,100$	$\,180 \pm 51 \pm \,89$
$T_3$	$2\,512 \pm 91 \pm \,42$	$\,85 \pm 77 \pm \,30$
2011	$95\,900 \pm 420 \pm 1\,100$	$3\,000 \pm 340 \pm 1\,140$

The total efficiencies within the fiducial volume include acceptance and trigger, reconstruction and selection efficiencies. The acceptance is defined by the selection placed on the particles in the final state: $p_{\text {T}} ^{\mu }>4.0$ GeV and $|\eta _{\mu }|<2.5$ for muons, $p_{\text {T}} ^{K}>1.0$ GeV and $\left| \eta _{K}\right| <2.5$ for kaons. In addition to the reweighting of the distributions of $p_{\text {T}} ^B$, $|\eta ^B|$ and the number of reconstructed PVs observed in data, the MC samples are reweighted according to the equivalent integrated luminosity associated with each trigger category and the Level-2 muon trigger algorithms used in 2012.

The trigger efficiencies are taken from a data-driven study based on the comparison of single-muon and dimuon triggers for events containing muon pairs from the decays of $J/\psi$ and $\Upsilon$ resonances [33]. Reconstruction and selection efficiencies are obtained from simulation. The signal selection requires the output of the continuum-BDT to be larger than 0.24.

**Table 4:** Values of the efficiency ratios $$R_{\varepsilon }^k$$ for the 2012 trigger categories and the 2011 sample, and their relative contributions to $$\mathcal {D}_{\mathrm {norm}}$$ (Eq. (2)). The first uncertainty is statistical and the second systematic. The systematic component includes the uncertainties from the MC reweighting and from data–MC discrepancies, as described in the text. The correction due to the $$B_s^0 $$ effective lifetime value discussed in the text is not applied to the numbers shown

Data category (*k*)	$R_{\varepsilon }^k$ = $(\varepsilon _{J/\psi K^+}/\varepsilon _{\mu ^+\mu ^-})_k$	Relative contribution to $\mathcal {D}_{\mathrm {norm}}$ (%)
$T_1$	$0.180 \pm 0.001\,\pm$ 0.009	68.3
$T_2$	$0.226 \pm 0.004\,\pm$ 0.014	6.0
$T_3$	$0.189 \pm 0.005\,\pm$ 0.022	3.5
2011	$0.156 \pm 0.002\,\pm$ 0.009	22.2

All efficiency terms are computed separately for the three trigger selections used in 2012 and for the 2011 sample. Table 4 provides the values of the efficiency ratios $R_{\varepsilon }^k$, for each of the categories ($k=1-4$), together with the statistical and systematic uncertainties described below.

The efficiency ratios shown in Table 4 are computed using the mean lifetime of $$B_s^0 $$ [25, 34] in the MC generator. The same efficiency ratios apply to the $$B^0_s \rightarrow \mu ^+\mu ^-$$ and $$B^0 \rightarrow \mu ^+\mu ^-$$ decays, within the MC statistical uncertainty of $${\pm }0.5~\%$$.

The statistical uncertainties in the efficiency ratios come from the finite number of events available for the simulated samples. The systematic uncertainty affecting $R_{\varepsilon }^k$ comes from four sources. A first contribution is due to the uncertainties in the DDW. This term is assessed from pseudo-MC studies, performed by varying the corrections within their statistical uncertainties. The RMS value of the distribution of $R_{\varepsilon }^k$ obtained from pseudo-MC samples is taken as the systematic uncertainty. The uncertainties range from ±1 to ±6 % depending on the category considered.

A second contribution is related to the trigger efficiencies. The effects of the statistical uncertainties in the data-driven efficiencies is evaluated with pseudo-MC studies, obtaining values in the range of ±1.5 to ±7 % in the different categories. An additional ±1.5 % uncertainty is added in quadrature for systematic effects. This term includes uncertainties in the Level-2 muon trigger algorithm, which are evaluated through data-driven studies performed using $J/\psi \, K^+$ and $\mu ^+\mu ^-$ candidates, and cancel to a large extent in the ratio of normalisation and signal channels.

A third source of systematic uncertainty arises from the differences between data and simulation observed in the modelling of the discriminating variables used in the continuum-BDT classifier (Table 2). For each of the 15 variables, the MC samples for $B^0_s \rightarrow \mu ^+\mu ^-$ and $B^+ \rightarrow J/\psi \,K^+$ are reweighted according to the distribution of the variable observed in $B^+ \rightarrow J/\psi \,K^+$ events from the data sample, after background subtraction. The isolation variable $I_{0.7}$ is computed using charged-particle tracks only, and differences between $B^+$ and $B_s^0$ are expected and were observed in previous studies [14]. Hence for this variable the reweighting procedure for the $B^0_s \rightarrow \mu ^+\mu ^-$ MC sample is based on $B^0_s \rightarrow J/\psi \, \phi$ data. For all discriminating variables but $I_{0.7}$, the value of the efficiency ratio is modified by less than 2 % by the reweighting procedure. For these variables, each variation is taken as an independent contribution to the systematic uncertainty in the efficiency ratio. For $I_{0.7}$ the reweighing procedure changes the efficiency ratio by $-5.3$ % for the 2012 data sample. A smaller effect is found for the 2011 sample, obtained with a different MC generator. Because of the significant mis-modelling, the 2012 MC samples obtained after reweighting on the distribution of $I_{0.7}$ are taken as a reference, thus correcting the central value of the efficiency ratio. The uncertainty in the correction is ${\pm }3.2$ % and is added to the sum in quadrature of the uncertainties assigned to the other discriminating variables. The total uncertainty in the modelling of the discriminating variables is the dominant contribution to the systematic uncertainties shown in Table 4.

A fourth source of systematic uncertainty arises from differences between the $B^0_s \rightarrow \mu ^+\mu ^-$ and the $B^+ \rightarrow J/\psi \,K^+$ channels related to the reconstruction efficiency of the kaon track and of the $B^+$ decay vertex [35]. These uncertainties are mainly related to inaccuracy in the modelling of passive material in the ID system and have been validated by studies performed on data. The corresponding systematic uncertainty is ${\pm }3.6$ %.

The efficiency ratios enter in Eq. (1) with the $\mathcal {D}_{\mathrm {norm}}$ term defined in Eq. (2). For each category *k*, the efficiency ratio is multiplied by the number of observed $B^+$ candidates and the trigger prescaling factor. The relative contributions of the $T_1$, $T_2$, $T_3$ and 2011 categories are shown in Table 4. The uncertainties in $R_{\varepsilon }^k$ are weighted accordingly and combined. For the trigger categories of the 2012 data sample, the correlations among the uncertainties due to DDW, trigger efficiency and mis-modelling of the discriminating variables are taken into account. Table 5 shows the different contributions and the total uncertainty on $\mathcal {D}_{\mathrm {norm}}$, equal to ${\pm }5.9$ %.

**Table 5:** Summary of the systematic uncertainties in the $\mathcal {D}_{\mathrm {norm}}$ term of Eq. (2)

Statistical uncertainty in simulation (%)	0.5
$p_{\text {T}}$, $\eta$ reweighting (%)	0.8
Trigger efficiency (%)	1.9
Data to MC discrepancy in discriminating variables (%)	4.2
$$K^+$$ and $$B^+ $$ reconstruction (%)	3.6
$B^+$ yield (%)	0.8
Total uncertainty (%)	5.9

A correction to the efficiency ratio for $B^0_s \rightarrow \mu ^+\mu ^-$ is needed because of the width difference $\Delta \Gamma _s$ between the $B_s^0$ eigenstates. According to the SM, the decay $B^0_s \rightarrow \mu ^+\mu ^-$ proceeds predominantly through the heavy state $B_{s,\mathrm {H}}$ [1, 15], which has width $\Gamma _{s,\mathrm {H}}= \Gamma _s - \Delta \Gamma _s/2$, i.e. $(6.2\pm 0.5)$ % smaller than the average $\Gamma _s$ [34]. The variation in the value of the $B^0_s \rightarrow \mu ^+\mu ^-$ mean lifetime was tested with simulation, and found to change the $B_s^0$ efficiency by $+4$ %, and consequently the $B_s^0$ to $B^+$ efficiency ratio. This correction is applied to the central value of $\mathcal {D}_{\mathrm {norm}}$ used in Sect. 12 for the determination of $\mathcal B$($B^0_s \rightarrow \mu ^+\mu ^-$).^2^ Due to the small value of $\Delta \Gamma _d$, no correction needs to be applied to the $B^0 \rightarrow \mu ^+\mu ^-$ decay.

### Comparison of normalisation yields with other measurements

The systematic acceptance and efficiencies uncertainties are minimised by using $B^+ \rightarrow J/\psi \,K^+$ as the normalisation channel and evaluating only efficiency ratios. However, event counts and absolute efficiency values for the reference channels can be used to extract the production cross sections for the purposes of comparisons with other measurements.

The yield of $B^+$ can be compared to the one obtained by ATLAS with 2.7 fb$^{-1}$ of data collected at $\sqrt{s}= 7$ TeV [35], and based on the same decay channel. In the comparison, the data collected at $\sqrt{s}= 8$ TeV for the present analysis were restricted to the phase space $p_{\text {T}} ^B>9.0$ GeV and $\left| \eta _B\right| <2.25$ used for the previous result. Trigger and preliminary selections are very similar, but the selections against continuum background and fake muons are used only in the present analysis. The difference in the collision energy is taken into account by comparing the measured production cross section to the prediction based on the fixed-order next-to-leading-log (FONLL) approximation [36]. The theoretical uncertainty in the extrapolation from 7 to 8 TeV is expected to be small compared to experimental uncertainties. The ratio of the observed to the predicted cross section was measured in Ref. [35] as $1.24\,\pm \,0.04\,\pm \,0.09$, where the statistical and systematic uncertainties include only the experimental ones. The corresponding value from the present analysis is $1.17\pm 0.02\pm 0.14$, with the uncertainty dominated by the systematic uncertainty in the efficiency of the continuum-BDT selection. The result is in agreement with the previous measurement. Correlated systematic uncertainties between the two analyses amount to $\pm 0.05$.

The measurements of $$B^0_s \rightarrow J/\psi \, \phi $$ and $$B^+ \rightarrow J/\psi \,K^+$$ yield, together with the corresponding acceptance and efficiency values, can be used to extract the production ratio $$B_s^0/B^+ $$, for $$10 \lesssim p_{\text {T}} ^B \lesssim 20$$ GeV and $$|\eta |<2.5$$, in *pp* collisions at $$\sqrt{s}= 8$$ TeV. Using world averages values [25] for the branching fractions to the final states, the resulting mean ratio of the hadronisation fractions $$f_s/f_u$$ is equal to $$0.236 \pm 0.014 \pm 0.018 \pm 0.021$$, where the first uncertainty is statistical, the second is the systematic uncertainty in the efficiency ratio and the third is the uncertainty in the branching fractions. The ratio is uniform across the kinematic range observed, and it varies by only $$-2~\%$$ if the $$B_s^0 $$ and $$B^+ $$ signals are extracted without applying the continuum-BDT selection. The normalisation procedure might not be free of bias, since the value of $$\mathcal{B}(B^0_s \rightarrow J/\psi \, \phi )$$ includes assumptions about $$f_s$$, and updating the assumptions may change it by about $$5~\%$$. The result nevertheless provides a satisfactory consistency check with the available measurements [37, 38]. The most direct comparison is with the recent value $$f_s/f_d = 0.240 \pm 0.020$$ [38], obtained by ATLAS from the analysis of 2.7 fb$$^{-1}$$ of data collected at $$\sqrt{s}= 7$$ TeV, and performed over the same $$p_{\text {T}} ^B$$ and $$\eta ^B$$ ranges used in this analysis. The uncertainty in that measurement is dominated by the prediction of the ratio of branching fractions $$\mathcal{B}(B^0_s \rightarrow J/\psi \, \phi )\,/\,\mathcal{B}(B^0 \rightarrow J/\psi K^{*0})$$. The ratio of the efficiency-corrected event yields observed at $$\sqrt{s}= 8$$ TeV in the present analysis can be compared to the corresponding value from Ref. [38] after rescaling by the ratio of branching fractions $$\mathcal{B}(B^+ \rightarrow J/\psi \,K^+)\, /\, [\mathcal{B}(B^0 \rightarrow J/\psi K^{*0})\!\times \! \mathcal{B}(K^{*0}\rightarrow K^+ \pi ^-)]$$, which is known with better accuracy than $$\mathcal{B}(B^0_s \rightarrow J/\psi \, \phi )$$. In this way, some systematic uncertainties are removed, and the ratio of the two results is $$0.96\pm 0.12$$. The largest contribution to the systematic uncertainty is from the efficiency of the continuum-BDT selection used in the present analysis.

In conclusion, the observed event rates for the normalisation channels are in agreement with previous measurements within uncertainties of about $12~\%$.

## Extraction of the signal yield

Dimuon candidates passing the preliminary selection and the multivariate selections against hadron misidentification and continuum background are classified according to three intervals in the continuum-BDT output: 0.240–0.346, 0.346–0.446 and 0.446–1. Each interval corresponds to an equal efficiency of $18~\%$ for signal events, and they are ordered according to increasing signal-to-background ratio. In each continuum-BDT interval, events from the four trigger and data categories are merged.

An unbinned extended maximum-likelihood fit is performed on the dimuon invariant mass distribution simultaneously across the three continuum-BDT intervals. The result of the fit is the total yield of $B^0_s \rightarrow \mu ^+\mu ^-$ and $B^0 \rightarrow \mu ^+\mu ^-$ events in the three BDT intervals. The parameters describing the background are allowed to vary freely and are determined by the fit. The fit model for signal and background is described in Sect. 11.1. The systematic uncertainties related to the BDT intervals, to the signal and to the background model are discussed in Sects. 11.1 and 11.2, and are included in the likelihood with Gaussian multiplicative factors with width equal to the systematic uncertainty.

### Signal and background model

The model for describing signal and background is based on simulations and on data collected in the mass sidebands of the search region.

**Fig. 6:**
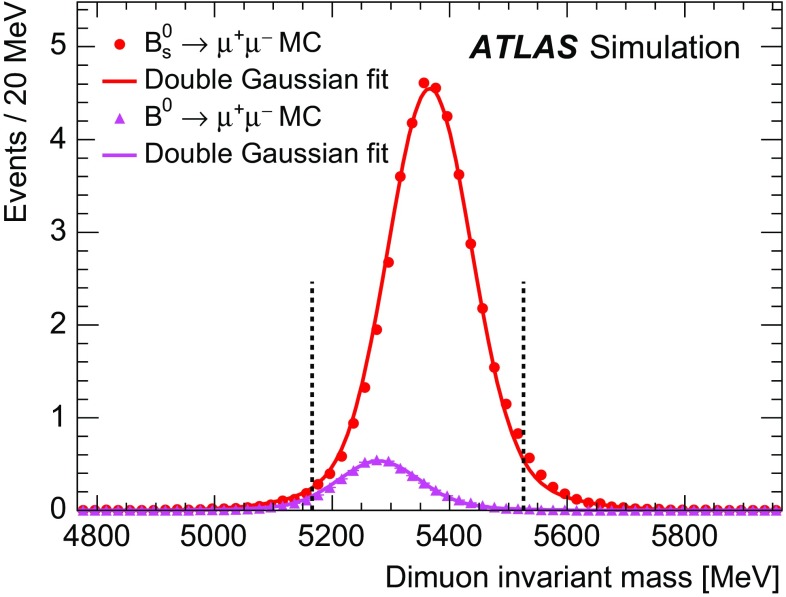
Dimuon invariant mass distribution for the $B_s^0$ and $B^0$ signals from simulation. The double Gaussian fits are overlaid. The two distributions are normalised to the SM prediction for the expected yield with an integrated luminosity of 25 fb$^{-1}$

The invariant mass distribution of the $B^0{}\!_{(s)} \rightarrow \mu ^+\mu ^-$ signal is described by a superposition of two Gaussian distributions, both centred at the $B^0$ or $B_s^0$ mass. The parameters are extracted from simulation, and they are taken to be uncorrelated with the BDT output. Systematic uncertainties in the mass scale and resolutions are considered separately. Figure 6 shows the invariant mass distributions for $B^0$ and $B_s^0$, obtained from MC events and normalised to the SM expectations.

The efficiency of the three intervals in the continuum-BDT output for $B^0{}\!_{(s)} \rightarrow \mu ^+\mu ^-$ events is calibrated with studies performed on the reference channels. The distribution of the BDT output is compared between MC and background-subtracted data. The differences observed in the ratio of data over simulation are described with a linear dependence on the BDT output. The slopes are equal within ${\pm }12$ % between $B^+ \rightarrow J/\psi \,K^+$ and $B^0_s \rightarrow J/\psi \, \phi$   and the mean value is used to reweight the BDT-output distribution in the $B^0{}\!_{(s)} \rightarrow \mu ^+\mu ^-$ MC sample. The corresponding absolute variations in the efficiencies are equal to $+1.8$ and $-1.8$ % respectively in the first and third BDT intervals. The values of the lower edge of the second and third BDT intervals are corrected in simulation to obtain equal efficiencies of 18.0 % in each interval.

The systematic uncertainties in the efficiency of the BDT intervals are obtained with a procedure similar to the one used for the event selection (Sect. 10). For each discriminating variable, the MC sample is reweighted according to the difference between simulation and data observed in the reference channels. The variation in the efficiency of each BDT interval is taken as the contribution to the systematic uncertainty due to mis-modelling of that variable. In each BDT interval, the sum in quadrature of the variations of all discriminating variables is found to be similar in the $B^+ \rightarrow J/\psi \,K^+$ and $B^0_s \rightarrow J/\psi \, \phi$ channels, and the average of the two is taken as the total systematic uncertainty in the efficiency. Absolute values of ${\pm }2.6$, ${\pm }1.0$ and ${\pm }2.3$ % are found respectively in the first, second and third interval. Gaussian terms are included in the likelihood in order to describe these uncertainties, taking care of constraining the sum of the efficiencies of the three intervals, since that uncertainty is already included in the selection efficiency.

**Fig. 7:**
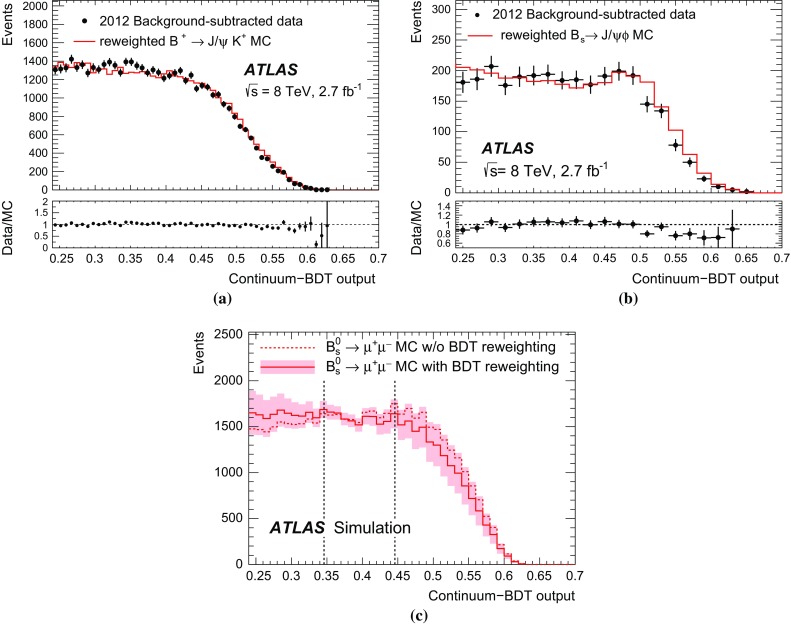
Data and MC distributions of $B^+ \rightarrow J/\psi \,K^+$ (**a**) and $B^0_s \rightarrow J/\psi \, \phi$ (**b**) for the continuum-BDT output and the MC distributions for $B^0_s \rightarrow \mu ^+\mu ^-$ (**c**). The MC samples are normalised to the number of data events. A linear correction has been applied to the MC distributions, equal for all channels, and a systematic uncertainty is assigned to the distribution of the $B^0{}\!_{(s)} \rightarrow \mu ^+\mu ^-$ MC sample, as discussed in the text and illustrated by the *dashed line* and the envelope shown in **c**. The *vertical dashed lines* in **c** correspond to the boundaries of the continuum-BDT intervals used for the signal extraction

Figure 7 shows the distribution of the continuum-BDT output from data and simulation for the reference channels, after reweighting the MC sample. The MC distribution for $B^0{}\!_{(s)} \rightarrow \mu ^+\mu ^-$ events is also shown, illustrating the correction based on the BDT output and the systematic uncertainty discussed above. The reweighting on the $I_{0.7}$ variables, discussed in Sect. 10 for the evaluation of the efficiency of the final event selection (BDT output $> 0.24$), is not applied to the events shown in Fig. 7, and in the evaluation of the relative efficiency of the intervals used for the extraction of the $B^0{}\!_{(s)} \rightarrow \mu ^+\mu ^-$ signal. Reweighting the BDT output is preferred over reweighting $I_{0.7}$, because of correlations present between the discriminating variables after the final selection is applied.

Finally, for the $B^0_s \rightarrow \mu ^+\mu ^-$ signal, the lifetime difference between $B_{s,\mathrm {H}}$ and $B_s^0$ requires further absolute corrections to the efficiency of the BDT intervals of $+0.3$ and $+1.8$ % respectively in the second and third interval.

The background is composed of the types of events described in Sect. 5: (a) the continuum background; (b) the background from partially reconstructed SS and SV events, which is present mainly in the low-mass sideband; (c) the peaking background.

The dependence of the continuum background on the dimuon invariant mass is described with a first-order polynomial. In the simulation, the slope of the distribution is similar in the three continuum-BDT intervals. The correlation between continuum-BDT and dimuon invariant mass is small, and similar between simulation and sideband data within large statistical uncertainties. Hence the slope of the mass dependence is described by independent parameters in the three intervals, subject to loose Gaussian constraints of uniformity within ${\pm }40~\%$ between the first and second interval, and ${\pm }80~\%$ between the first and the third. Such variations of slope are larger than those observed in simulation, and consistent with those determined from data. Deviations from these assumptions are discussed below in Sect. 11.2. The normalisation of the continuum background is also extracted independently in each BDT interval.

The SS+SV background has a dimuon invariant mass distribution peaking below the low-mass sideband region. The mass dependence is derived from data in the low-mass sideband region, and described with an exponential function with equal shape in the three continuum-BDT intervals. The value of the shape parameter is extracted from the fit to data. The normalisation values are extracted independently in each interval.

The invariant mass distribution of the peaking background is very similar to the $B^0$ signal, as shown in Fig. 1b. In the fit, this contribution is included with fixed mass shape and with a normalisation of $1.0\;\pm \;0.4$ events, as discussed in Sect. 6. This contribution is equally distributed among the three intervals of continuum-BDT.

The fitting procedure is tested with pseudo-MC experiments, as discussed below. The use of three intervals in the continuum-BDT output is found to optimise the performance of the likelihood fit, with all BDT intervals contributing to the determination of the background, while the second and in particular the third interval provide sensitivity to the signal yield.

### Systematic uncertainties in the fit

Studies based on pseudo-MC experiments are used to assess the sensitivity of the fit to the input assumptions. Variations in the description of signal and background components are used in the generation of the pseudo-MC samples. The corresponding deviations in the average numbers $N_s$, $N_d$ of $B_s^0$ and $B^0$ events returned by the fit, run in the nominal configuration, are taken as systematic uncertainties. The amplitude of the variations in the generation of the pseudo-MC samples is determined in some cases by known characteristics of the ATLAS detector (reconstructed momentum scale and momentum resolution), in others using MC evaluation (background due to semileptonic three-body $B^0{}\!_{(s)}$ decays and to $B_c \rightarrow J/ \psi \mu$), and in others from uncertainties determined from data in the sidebands and from simulation (shapes of the background components and their variation across the continuum-BDT intervals).

**Fig. 8:**
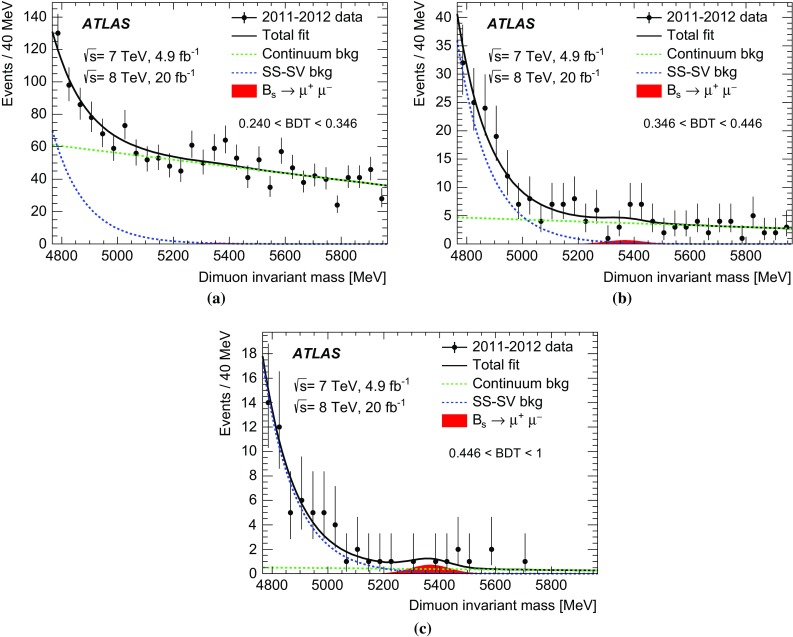
Dimuon invariant mass distributions in the unblinded data, in the three intervals of continuum-BDT output. Superimposed is the result of the maximum-likelihood fit, obtained imposing the boundary of non-negative signal contributions. The total fit is shown as a *black continuous line*, the *filled area* corresponds to the observed signal component, the *blue dashed line* to the SS+SV background, and the *green dashed line* to the continuum background

The pseudo-MC experiments were generated with the normalisation of the continuum and SS+SV components obtained from the fit to the data in the sideband of the invariant mass distribution, and the peaking background from the expectation discussed in Sect. 6. The signal was generated with different configurations, corresponding to the SM prediction, to smaller values of $\mathcal B$($B^0_s \rightarrow \mu ^+\mu ^-$) and to smaller/larger values of $\mathcal B$($B^0 \rightarrow \mu ^+\mu ^-$).

For all variations in the assumptions and all configurations of the signal amplitudes, the distributions of the differences between results and generated values, divided by the fit errors (pull distributions), are found to be correctly described by Gaussian functions with widths approximately equal to one and values of the mean smaller than 0.2 for $B^0_s \rightarrow \mu ^+\mu ^-$ and smaller than 0.4 for $B^0 \rightarrow \mu ^+\mu ^-$. The distributions obtained from pseudo-MC samples generated according to the nominal fit model are used to evaluate fit biases. For $B^0_s \rightarrow \mu ^+\mu ^-$ the fit bias is negligible. For $B^0 \rightarrow \mu ^+\mu ^-$ the bias on the yield is smaller than 25 % of the fit error, and it is included as an additional systematic uncertainty.

The shifts in $N_s$ or $N_d$ are combined by considering separately the sums in quadrature of the positive and negative shifts and taking the larger as the symmetric systematic uncertainty. For $B_s^0$, the total systematic uncertainty is found to increase with the assumed size of the signal, with a dependence $\sigma _\mathrm {syst} (N_s) = \sqrt{2^2 + (0.06\times N_s)^2}$. The total systematic uncertainty for $B^0$ is approximately $\sigma _\mathrm {syst} (N_d) = 3$. Most of the shifts observed have opposite sign for $N_s$ and $N_d$, resulting in a combined correlation coefficient in the systematic uncertainties of $\rho _\mathrm {syst}=-0.7$.

The fit to the yield of $B_s^0$ and $B^0$ events is modified by including in the likelihood two smearing parameters for $N_s$ and $N_d$ that are constrained by a combined Gaussian distribution parameterised by the values of $\sigma _\mathrm {syst} (N_s)$, $\sigma _\mathrm {syst} (N_d)$ and $\rho _\mathrm {syst}$.

### Results of the signal yield extraction

Including both the 2012 and 2011 data-taking periods, the numbers of background events contained in the signal region (5166–5526 MeV) are computed from the interpolation of the data observed in the sidebands. The values $509\pm 28$, $32\pm 6$ and $4.8\pm 1.9$ events are obtained respectively in the three intervals of continuum-BDT. For comparison, the total expected number of signal events according to the SM prediction is 41 and 5 respectively for $N_s$ and $N_d$, equally distributed among the three intervals.^3^

Once the signal region is unblinded, a total of 1951 events in the full mass range of 4766–5966 MeV are used for the likelihood fit to signal and background. Without applying any boundary on the values of the fitted parameters, the values determined by the fit are $N_s = 16 \pm 12$ and $N_d = - 11 \pm 9$, where the uncertainties correspond to likelihood variations of $- 2\,\Delta \ln (L) = 1$. The likelihood includes the systematic uncertainties discussed above, but statistical uncertainties largely dominate. The primary result of this analysis is obtained by applying the natural boundary of non-negative yields, for which the fit returns the values $N_s = 11$ and $N_d = 0.$ The uncertainties in the result of the fit are discussed in Sect. 12, where the measured values of the branching fractions are presented.

Figure 8 shows the dimuon invariant mass distributions in the three intervals of continuum-BDT, together with the projections of the likelihood fit.

For comparison, the value $N_d$ can be constrained according to the SM expectation for the ratio $\mathcal{B}(B^0 \rightarrow \mu ^+\mu ^-)/\mathcal{B}(B^0_s \rightarrow \mu ^+\mu ^-)$ [1] multiplied by the ratio of the hadronisation probabilities $f_d/f_s$ [38], rather than being extracted independently from the fit. In this case the value of $N_s$ changes by $-0.8$, while $N_d = N_\mathrm {s}/8.3 \approx 1.2$.

## Branching fraction extraction

The branching fractions for the decays $B^0_s \rightarrow \mu ^+\mu ^-$ and $B^0 \rightarrow \mu ^+\mu ^-$ are extracted from data using a profile-likelihood fit. The likelihood is obtained from the one used for $N_s$ and $N_d$ replacing the fit parameters with the corresponding branching fractions divided by normalisation terms in Eq. (1), and including Gaussian multiplicative factors for the normalisation uncertainties.

The normalisation terms include external inputs for the $$B^+ $$ branching fraction and the relative hadronisation probability. The first is obtained from world averages [25] as the product of $$\mathcal{B}(B^+ \rightarrow J/\psi \,K^+)=(1.027 \pm 0.031) \times 10^{-3}$$ and $$\mathcal{B}(J/\psi \rightarrow \mu ^{+} \mu ^{-})=(5.961 \pm 0.033)~\%$$. The second is equal to one for $$B^0 $$, while for $$B_s^0 $$ it is taken from the ATLAS measurement $$f_s/f_d = 0.240 \pm 0.020$$ [38], assuming $$f_u/f_d=1$$ [34].

The efficiency- and luminosity-weighted number of events for the normalisation channel enters in Eq. (1) with the denominator $$\mathcal {D}_{\mathrm {norm}}$$ (Eq. (2)). The values $$\mathcal {D}_{\mathrm {norm}} =(2.88 \pm 0.17) \times 10^{6}$$ for $$B_s^0 $$ and $$(2.77 \pm 0.16) \times 10^{6}$$ for $$B^0 $$ are obtained using Tables 3 and 4 for each category, together with the combined uncertainty from Table 5, and including the $$+4$$ % correction to the $$B^0_s \rightarrow \mu ^+\mu ^-$$ efficiency due to the lifetime difference between $$B_{s,\mathrm {H}}$$ and $$B_s^0 $$.

The combination of $$B^+ $$ branching fraction, hadronisation probabilities and $$\mathcal {D}_{\mathrm {norm}}$$, i.e. the single-event sensitivity, is equal to $$(8.9\pm 1.0)\times 10^{-11}$$ for $$B^0_s \rightarrow \mu ^+\mu ^-$$ and $$(2.21\pm 0.15)\times 10^{-11}$$ for $$B^0 \rightarrow \mu ^+\mu ^-$$.

The values of the branching fractions that maximise the profile-likelihood within the constraint of non-negative values are $\mathcal{B}(B^0_s \rightarrow \mu ^+\mu ^-) = 0.9 \times 10^{-9}$ and $\mathcal{B}(B^0 \rightarrow \mu ^+\mu ^-) = 0$. That constraint is applied for all results discussed in this section if not otherwise stated.

A Neyman construction [39] is used to determine the 68.3 % confidence interval for $\mathcal B$($B^0_s \rightarrow \mu ^+\mu ^-$) with pseudo-MC experiments, obtaining:

$$\begin{aligned} \mathcal{B}(B^0_s \rightarrow \mu ^+\mu ^-) = \left( 0.9^{+1.1}_{-0.8} \right) \times 10^{-9}. \end{aligned}$$

The uncertainties include both the statistical and systematic contributions. The two components are separated by repeating the likelihood fit after setting all systematic uncertainties to zero. The statistical uncertainty is dominant, with the systematic uncertainty equal to $\pm \,0.3\times 10^{-9}$.

The observed significance of the $B^0_s \rightarrow \mu ^+\mu ^-$ signal is determined from pseudo-MC experiments, with a hypothesis test based on the likelihood ratio $-\ln [L(\mathrm {no\!-\!signal})/L(\mathrm {max})]$ [40], and is equal to 1.4 standard deviations. For this test, $\mathcal B$($B^0 \rightarrow \mu ^+\mu ^-$) is left free to be determined in the fit. The corresponding expected significance is 3.1 standard deviations for the SM predictions $\mathcal{B}(B^0_s \rightarrow \mu ^+\mu ^-) = (3.65 \pm 0.23) \times 10^{-9}$ and $\mathcal{B}(B^0 \rightarrow \mu ^+\mu ^-) = (1.06 \pm 0.09) \times 10^{-10}$ [1].

Pseudo-MC experiments are also used to evaluate the compatibility of the observation with the SM prediction. A hypothesis test based on $-\ln [L(\mathrm {SM})/L(\mathrm {max})]$ is performed for the simultaneous fit to $\mathcal B$($B^0_s \rightarrow \mu ^+\mu ^-$) and $\mathcal B$($B^0 \rightarrow \mu ^+\mu ^-$). The result is $p=0.048\pm 0.002$, corresponding to 2.0 standard deviations.

**Fig. 9:**
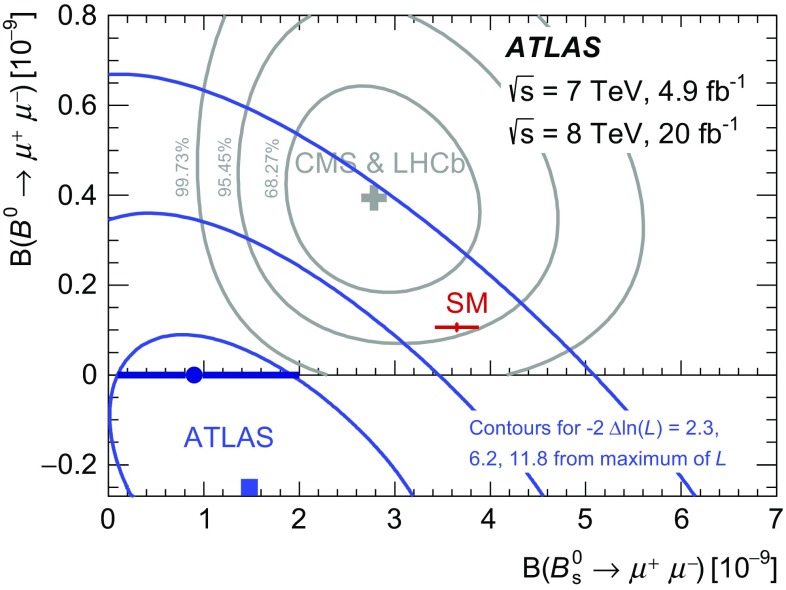
Contours in the plane $\mathcal{B}(B^0_s \rightarrow \mu ^+\mu ^-), \mathcal{B}(B^0 \rightarrow \mu ^+\mu ^-)$ for intervals of $-2\, \Delta \ln (L)$ equal to 2.3, 6.2 and 11.8 relative to the absolute maximum of the likelihood, without imposing the constraint of non-negative branching fractions. Also shown are the corresponding contours for the combined result of the CMS and LHCb experiments, the SM prediction, and the maximum of the likelihood within the boundary of non-negative branching fractions, with the *error bars* covering the 68.3 % confidence range for $\mathcal B$($B^0_s \rightarrow \mu ^+\mu ^-$)

Figure 9 shows the contours in the plane of $\mathcal B$($B^0_s \rightarrow \mu ^+\mu ^-$) and $\mathcal B$($B^0 \rightarrow \mu ^+\mu ^-$) drawn for values of $-2\, \Delta \ln (L)$ equal to 2.3, 6.2 and 11.8, relative to the maximum of the likelihood, allowing negative values of the branching fractions. The maximum within the physical boundary is shown with error bars indicating the 68.3 % interval for the value of $\mathcal B$($B^0_s \rightarrow \mu ^+\mu ^-$). Also shown are the corresponding contours obtained in the combination of the results of the CMS and LHCb experiments[13], and the prediction based on the SM.

Using the CL$_\mathrm {s}$ method [41] implemented with pseudo-MC experiments, an upper limit is placed on the $B^0_s \rightarrow \mu ^+\mu ^-$ branching fraction at the 95 % confidence level:

$$\begin{aligned} \mathcal{B}(B^0_s \rightarrow \mu ^+\mu ^-) < 3.0\times 10^{-9}\, (95~\% \, \mathrm {CL}). \end{aligned}$$

The limit is obtained under the hypothesis of background only, with $\mathcal B$($B^0 \rightarrow \mu ^+\mu ^-$) left free to be determined in the fit. The expected limit is $1.8^{+0.7}_{-0.4} \times 10^{-9}$.

An upper limit based on the CL$$_\mathrm {s}$$ method is also set on $$\mathcal B$$($$B^0 \rightarrow \mu ^+\mu ^-$$). The expected limit obtained from pseudo-MC samples generated according to the observed amplitudes of backgrounds and $$B_s^0 $$ signal is $$\left( 5.7^{+2.1}_{-1.5} \right) \times 10^{-10}$$ at a confidence level of 95 %. The observed limit is:

$$\begin{aligned} \mathcal{B}(B^0 \rightarrow \mu ^+\mu ^-) < 4.2\times 10^{-10}\, (95~\% \, \mathrm {CL}). \end{aligned}$$

The observed upper limit is above the SM prediction and also covers the central value of the combination of the measurements by CMS and LHCb [13]. The expected significance for $\mathcal B$($B^0 \rightarrow \mu ^+\mu ^-$) according to the SM prediction is equal to 0.2 standard deviations.

## Conclusions

A study of the rare decays of $B_s^0$ and $B^0$ mesons into oppositely charged muon pairs is presented, based on 25 fb$^{-1}$ of 7 and 8 TeV proton–proton collision data collected by the ATLAS experiment in Run 1 of LHC.

For $B^0$ an upper limit $\mathcal{B}(B^0 \rightarrow \mu ^+\mu ^-) < 4.2 \times 10^{-10}$ is placed at the 95 % confidence level, based on the CL$_\mathrm {s}$ method. The limit is compatible with the predictions based on the SM and with the combined result of the CMS and LHCb experiments.

For $B_s^0$ the result is $\mathcal{B}(B^0_s \rightarrow \mu ^+\mu ^-) = \left( 0.9^{+1.1}_{-0.8} \right) \times 10^{-9}$, where the uncertainty includes both the statistical and systematic components. An upper limit $\mathcal{B}(B^0_s \rightarrow \mu ^+\mu ^-) < 3.0 \times 10^{-9}$ at 95 % CL is placed, lower than the SM prediction, and in better agreement with the measurement of CMS and LHCb.

A *p* value of 4.8 % is found for the compatibility of the results with the SM prediction.

## Footnotes

1 ATLAS uses a right-handed coordinate system with its origin at the nominal interaction point. The *z*-axis is along the beam pipe, the *x*-axis points to the centre of the LHC ring and the *y*-axis points upward. Cylindrical coordinates $(r,\phi )$ are used in the transverse plane, *r* being the distance from the origin and $\phi$ being the azimuthal angle around the beam pipe. The pseudorapidity $\eta$ is defined as $\eta = - \mathrm{ln}[\mathrm{tan}(\theta /2)]$ where $\theta$ is the polar angle.

2 The decay time distribution of $B^0_s \rightarrow \mu ^+\mu ^-$ is predicted to be different from the one of $B_{s,\mathrm {H}}$ in scenarios of new physics, with the effect related to the observable $\mathcal {A}^{\,\mu \mu }_{\,\Delta \Gamma }$ [15, 16]. The maximum possible deviation from the SM prediction of $\mathcal {A}^{\,\mu \mu }_{\,\Delta \Gamma }=+1$, is for $\mathcal {A}^{\,\mu \mu }_{\,\Delta \Gamma }=-1$, for which the decay time distribution of $B^0_s \rightarrow \mu ^+\mu ^-$ corresponds to the distribution of the $B_{s,\mathrm {L}}$ eigenstate. In the comparison with new physics predictions, the value of $\mathcal B$($B^0_s \rightarrow \mu ^+\mu ^-$) obtained from this analysis should be corrected by $+4~\% \times (1- \mathcal {A}^{\,\mu \mu }_{\,\Delta \Gamma })$.

3 The values of the single-event sensitivity are discussed in Sect. 12.
